# Near-infrared-triggered photon upconversion tuning in all-inorganic cesium lead halide perovskite quantum dots

**DOI:** 10.1038/s41467-018-05947-2

**Published:** 2018-08-27

**Authors:** Wei Zheng, Ping Huang, Zhongliang Gong, Datao Tu, Jin Xu, Qilin Zou, Renfu Li, Wenwu You, Jean-Claude G. Bünzli, Xueyuan Chen

**Affiliations:** 1 CAS Key Laboratory of Design and Assembly of Functional Nanostructures, and Fujian Key Laboratory of Nanomaterials, Fujian Institute of Research on the Structure of Matter, Chinese Academy of Sciences, Fuzhou, 350002 Fujian China; 2 University of Chinese Academy of Sciences, 100049 Beijing, China; 3 Institute of Chemical Sciences and Engineering, Swiss Federal Institute of Technology, Lausanne (EPFL), CH-1015 Lausanne, Switzerland

## Abstract

All-inorganic CsPbX_3_ (X = Cl, Br, and I) perovskite quantum dots (PeQDs) have shown great promise in optoelectronics and photovoltaics owing to their outstanding linear optical properties; however, nonlinear upconversion is limited by the small cross-section of multiphoton absorption, necessitating high power density excitation. Herein, we report a convenient and versatile strategy to fine tuning the upconversion luminescence in CsPbX_3_ PeQDs through sensitization by lanthanide-doped nanoparticles. Full-color emission with wavelengths beyond the availability of lanthanides is achieved through tailoring of the PeQDs bandgap, in parallel with the inherent high conversion efficiency of energy transfer upconversion under low power density excitation. Importantly, the luminescent lifetimes of the excitons can be enormously lengthened from the intrinsic nanosecond scale to milliseconds depending on the lifetimes of lanthanide ions. These findings provide a general approach to stimulate photon upconversion in PeQDs, thereby opening up a new avenue for exploring novel and versatile applications of PeQDs.

## Abstract (web summary)

Optimizing luminescence from quantum dots benefits various optoelectronic and photovoltaic applications. Here the authors drive high-efficiency, tunable upconversion luminescence in perovskite quantum dots by energy transfer from lanthanide-doped nanoparticles excited by near-infrared light, to produce full-color emission with low driving power.

## Introduction

All-inorganic cesium lead halide (CsPbX_3_, X = Cl, Br, and I) perovskite quantum dots (PeQDs) have recently attracted intensive attention in a wide array of research fields, owing to their outstanding physicochemical properties such as large absorption coefficients, high photoluminescence (PL) quantum yields (QYs), narrow emission bands, and tunable bandgap and PL emissions by varying the halide composition^1–4^. These elegant characteristics make CsPbX_3_ PeQDs ideal candidates as light-harvesting or light-emitting materials for diverse optoelectronic and photovoltaic applications^5–12^. Although PeQDs exhibit excellent linear optical properties under ultraviolet (UV) or visible light excitation, their nonlinear optical properties such as near-infrared (NIR)-triggered photon upconversion are limited by low efficiency ( < 10^−8^) of multiphoton absorption and the requirement of expensive pulsed lasers for excitation^13–18^. In contrast to linear absorption and emission, the nonlinear upconversion analogs feature a series of advantages including a large penetration depth, high spatial resolution, minimal background interference, and little damage to the targeted samples, which hold great promise in areas as diverse as multiplexed optical encoding, three-dimensional displays, super-resolution bioimaging, and effective solar spectrum conversion^19–26^.

As compared with PeQDs, lanthanide-doped nanoparticles (NPs) are much more efficient (10^−1^–10^−3^) in photon upconversion through successive photon absorption and energy transfer processes within lanthanide ions, and thus can be excited by using a low-cost continuous-wave (CW) diode laser^27–30^. By controlling the energy transfer processes based on core/shell engineering, these NPs can produce efficient upconversion luminescence (UCL) with wavelengths spanning from UV to NIR and lifetimes ranging from microseconds to milliseconds^31–36^. Nevertheless, upconversion tuning in lanthanide-doped NPs requires cumbersome experimentation involving repeated trials to optimize the dopant concentration^37^. Moreover, there are inherent limitations on wavelength tunability in lanthanide-doped NPs due to the defined and discrete energy levels of lanthanide ions.

To circumvent these limitations, it is essential to bring together both advantages of lanthanide-doped NPs and PeQDs, whereby lanthanide-doped NPs may serve as an effective sensitizer for PeQDs to boost their upconversion efficiency, whereas PeQDs with continuously tunable emission bands could extend the emission wavelengths of lanthanide-doped NPs. Unfortunately, previous endeavors on this aspect through non-radiative Förster resonance energy transfer (FRET) from lanthanide-doped NPs to quantum dots usually resulted in serious quenching of overall UCL intensity and negligibly weak quantum dots emissions, because it is notoriously difficult to control the number of energy acceptors in close proximity of the donors within an effective FRET distance^38–40^.

**Fig. 1:**
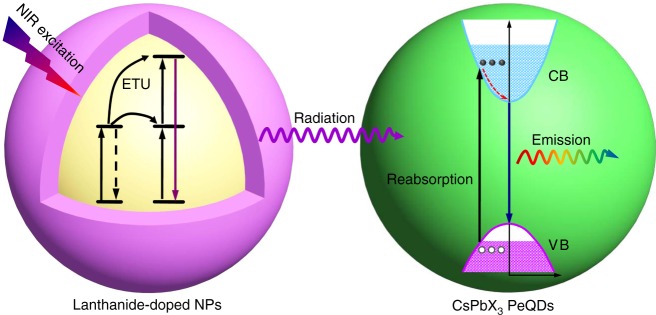
Schematic representation of the radiative energy transfer upconversion (RETU) processes in all-inorganic CsPbX_3_ perovskite quantum dots (PeQDs) through sensitization by lanthanide-doped nanoparticles (NPs). Lanthanide-doped NPs function as the energy donor to convert the NIR excitation light into the ultraviolet (UV) and visible emission light through successive photon absorption and energy transfer upconversion (ETU) processes within lanthanides. The emission light from the NPs is then reabsorbed by PeQDs to create electron–hole pairs (excitons) in the conduction band (CB) and valence band (VB), followed by photon emission through exciton recombination. The solid and dash lines represent the electronic transitions

Herein, we report a convenient and versatile approach to fine tuning the UCL in CsPbX_3_ PeQDs through sensitization by lanthanide-doped NPs. We demonstrate that the sensitization is governed by a radiative energy transfer upconversion (RETU) process. In our design (Fig. 1), lanthanide-doped NPs function as the energy donor to convert the NIR excitation light into the UV and visible emission light through successive photon absorption and energy transfer upconversion processes within lanthanides. The emission light from the NPs is then reabsorbed by PeQDs to create electron–hole pairs (excitons) in the conduction band and valence band, followed by photon emission through exciton recombination. A key feature of RETU is that the PeQDs possess large absorption coefficient and high PLQY. As a result, tunable upconversion emission with wavelengths beyond the availability of lanthanide-doped NPs is likely to be realized via bandgap tailoring of PeQDs, in parallel with the benefits of lifetime tunability by lanthanide ions and the inherent high conversion efficiency of energy transfer upconversion under low power density excitation.

## Results

### Synthesis and characterization

**Fig. 2:**
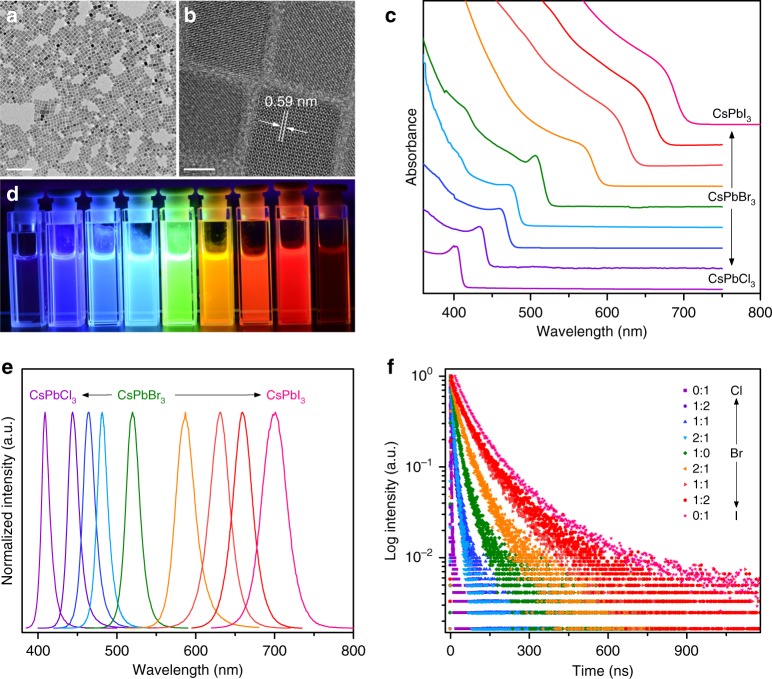
Synthesis and characterization of CsPbX_3_ perovskite quantum dots (PeQDs). **a** Transmission electron microscopy (TEM) and **b** high-resolution TEM images of the as-synthesized CsPbBr_3_ PeQDs. The scale bars in (**a**) and (**b**) represent 100 nm and 5 nm, respectively. **c** Optical absorption spectra, **d** photoluminescence (PL) photograph (λ = 365 nm), **e** PL spectra (λ = 360 nm) and **f** PL decays of the as-synthesized CsPbX_3_ PeQDs with varying halide compositions

High-quality CsPbX_3_ PeQDs were synthesized through a facile and novel method by injecting HX (X = Cl, Br, and I) to the precursor solution of Cs-oleate and Pb(II)-oleate at an elevated temperature (Methods). Structure and morphology characterizations through transmission electron microscopy (TEM), X-ray powder diffraction, energy dispersive X-ray spectra, and selected area electron diffraction revealed cubic phase and high crystallinity of the PeQDs with particle sizes of 11.0–13.3 nm (Fig. 2a, b and Supplementary Figs. 1 and 2). Optical absorption spectra showed that the PeQDs had large absorbance in the UV and visible spectral region with band edges shifting from 410 nm to 700 nm as the halide composition changed from Cl^−^ to I^−^ (Fig. 2c), indicating bandgap tailoring of PeQDs by adjusting the halide composition^41^. Upon UV excitation at 365 nm, these PeQDs displayed remarkably bright PL with full-gamut color tuning from violet to green and deep red (Fig. 2d). PL spectra of the PeQDs exhibited tunable emission band, which can be ascribed to the band-edge exciton recombination over the entire visible spectral region^42^, with bandwidths of 11.7–37.0 nm (Fig. 2e and Supplementary Table 1). PL decays indicated effective PL lifetimes of the band-edge excitons in the range of 1.8–81.1 ns with faster decay for wider-bandgap PeQDs (Fig. 2f and Supplementary Table 2). The absolute PLQYs, defined as the ratio of the number of emitted photons to the number of absorbed photons, were determined to be 26.0–79.8% with the highest value of 79.8% for CsPbBr_3_ PeQDs (Supplementary Table 1).

**Fig. 3:**
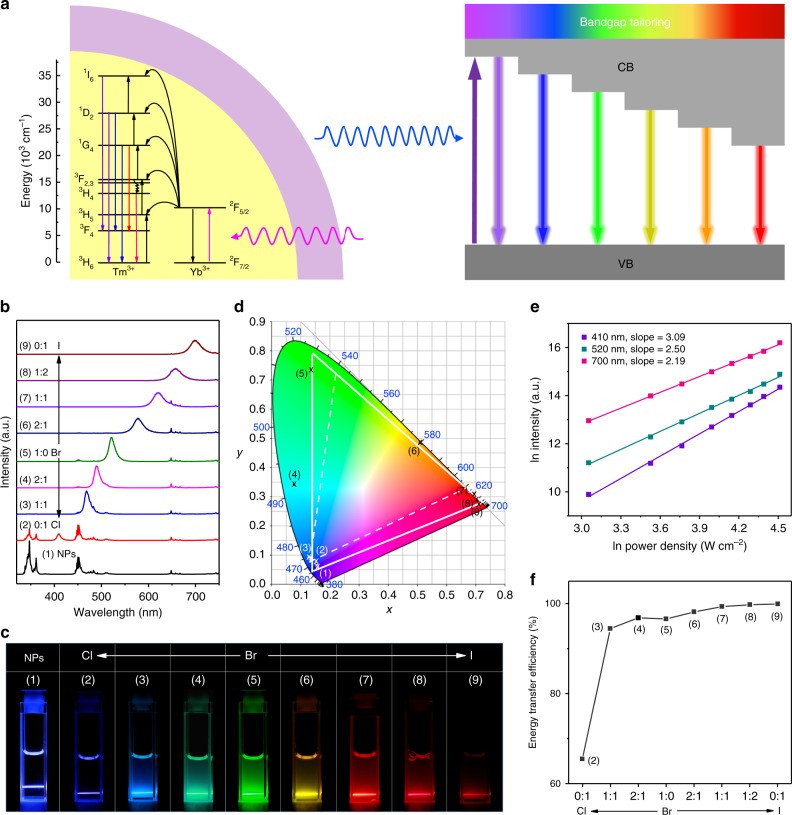
Full-color upconversion tuning in CsPbX_3_ perovskite quantum dots (PeQDs) through sensitization by lanthanide-doped nanoparticles (NPs). **a** Simplified energy-level scheme of LiYbF_4_:0.5%Tm^3+^@LiYF_4_ core/shell NPs indicating major upconversion processes, and schematic illustration of full-color upconversion tuning in CsPbX_3_ PeQDs through sensitization by the NPs. Black lines represent non-radiative energy transfer and the curved arrows denote internal conversion. **b** Upconversion luminescence (UCL) spectra for LiYbF_4_:0.5%Tm^3+^@LiYF_4_ core/shell NPs and the NP-sensitized CsPbX_3_ PeQDs (NPs: 1 mg mL^−1^; PeQDs: 2 mg mL^−1^) with varying halide compositions under 980 nm continuous-wave (CW) diode laser excitation at a power density of 50 W cm^−2^. **c** Photographs of samples (1–9) under 980 nm illumination, showing color tuning through bandgap tailoring of PeQDs. **d** Corresponding color gamut of the emission colors (solid white triangle) from the samples shown in (**c**), compared with the color gamut (dashed white triangle) defined in the NTSC television color standard. **e** Double logarithmic plots of intensity vs. excitation power for the upconverted exciton emissions from CsPbCl_3_, CsPbBr_3_, and CsPbI_3_ PeQDs at 410, 520, and 700 nm, respectively. **f** Calculated energy transfer efficiency in NP-sensitized PeQDs, as obtained from (**b**)

We synthesized LiYbF_4_:Tm^3+^@LiYF_4_ core/shell NPs through a high-temperature co-precipitation method (Supplementary Note 1) and selected them as the energy donors in view of their intense upconversion emission at 362 nm that can potentially excite the whole series of CsPbX_3_ PeQDs^43^. The as-synthesized NPs were rhombohedral and highly crystallized with a mean size of (26.7 ± 1.2) × (33.3 ± 1.8) nm and a shell thickness of 5.5 ± 0.6 nm (Supplementary Fig. 3). Under 980 nm excitation, the NPs displayed intense UCL with a set of sharp and typical emission peaks from Tm^3+^, which were divided into four regions: the UV region with peaks at 347 nm (^1^I_6_ → ^3^F_4_) and 362 nm (^1^D_2_ → ^3^H_6_), the blue region with peaks at 450 nm (^1^D_2_ → ^3^F_4_) and 483 nm (^1^G_4_ → ^3^H_6_), the red region with single peak at 648 nm (^1^G_4_ → ^3^F_4_), and the NIR region with a peak at 792 nm (^3^H_4_ → ^3^H_6_) (see Fig. 3a for a simplified diagram of transitions in the Yb^3+^–Tm^3+^ system and Supplementary Fig. 4)^43^. Power dependence investigations revealed that at least four pump photons are needed to populate the ^1^D_2_ level of Tm^3+^ to yield the UV emissions and three pump photons are required to feed the ^1^G_4_ level to produce the blue and red emissions (Supplementary Fig. 5)^44^. The UCL lifetimes were determined to be 217, 253, 447, and 601 μs for the decays from ^1^I_6_, ^1^D_2_, ^1^G_4_, and ^3^H_4_ of Tm^3+^, respectively (Supplementary Fig. 6).

### Full-color upconversion tuning in NP-sensitized CsPbX_3_ PeQDs

To sensitize photon upconversion in CsPbX_3_ PeQDs, we dispersed the PeQDs with LiYbF_4_:0.5%Tm^3+^@LiYF_4_ core/shell NPs in a homogeneous colloidal cyclohexane solution with a weight ratio of 2:1, in which the NPs can function as an internal UV or blue lamp to illuminate the PeQDs by utilizing the intense UCL of Tm^3+^ (Fig. 3a). Figure 3b shows the NP-sensitized UCL spectra for CsPbX_3_ PeQDs under 980 nm CW diode laser excitation. Intriguingly, we observed that the characteristic band-edge exciton emissions from the PeQDs were dominant in the UCL spectra, whereas the emissions of Tm^3+^ from the NPs were selectively quenched in accordance with the PeQDs absorption, which indicates an efficient energy transfer from the NPs to PeQDs as CW laser cannot trigger photon upconversion in pure PeQDs (Supplementary Fig. 7)^17^. As a result, multicolor emission with color gamut beyond the three primary colors can be easily achieved by varying the halide composition in PeQDs (Fig. 3c). Moreover, because of the narrow bandwidths of the PeQDs emissions, highly saturated Red-Green-Blue colors can be generated, leading to a wide color gamut with a triangle in the Commission Internationale de l'Eclairage chromaticity diagram encompassing 140% of that defined in the National Television System Committee (NTSC) color standard (Fig. 3d and Supplementary Table 3)^45^. Such a wide color gamut tuned by NP-sensitized PeQDs is inaccessible by either lanthanide-doped NPs or PeQDs alone and is highly desired for optical coding and three-dimensional displays^46–48^.

Power dependence investigations reveal that the slope of the double logarithmic plots of intensity vs. excitation power is larger than 3 for the upconverted exciton emission from CsPbCl_3_ and larger than 2 for the emissions from CsPbBr_3_, CsPbI_3_, and mixed CsPb(Cl/Br)_3_ and CsPb(Br/I)_3_ (Fig. 3e and Supplementary Fig. 8). According to Pollnau et al.^34^, this may be interpreted as reflecting four-photon and three-photon processes, respectively, whereby energy transfer upconversion appears to be the dominant mechanism. It implies that, at least four pump photons are required to generate the UV emissions of Tm^3+^ to excite CsPbCl_3_, whereas only three pump photons are necessary to produce the blue or red emissions of Tm^3+^, which, in turn, sensitize CsPbBr_3_, CsPbI_3_, CsPb(Cl/Br)_3_, or CsPb(Br/I)_3_ PeQDs. These results show unambiguously that the upconverted exciton emissions in NP-sensitized PeQDs originate from the NPs-to-PeQDs energy transfer. As shown in Fig. 3f, the energy transfer efficiency was calculated to increase from 65.5 to 96.6 and 99.9% as the halide composition changed from Cl^−^ to Br^−^ and I^−^ (see Supplementary Note 2 and Supplementary Table 4 for details in definition and calculation). The lower efficiency of energy transfer from the NPs to CsPbCl_3_ can be attributed to the smaller absorbance of CsPbCl_3_ in UV than the other CsPbX_3_ PeQDs, as evidenced by their absorption spectra. The absolute upconversion QYs, upon excitation at 980 nm with a power density of 100 W cm^−2^, were determined to be 0.06 ± 0.01%, 0.39 ± 0.08%, and 0.36 ± 0.09% for exciton emissions in NP-sensitized CsPbCl_3_, CsPbBr_3_, and CsPbI_3_ PeQDs, respectively (Supplementary Note 3 and Supplementary Table 5). The overall upconversion QYs of the NP-sensitized PeQDs are in the range of 0.33–0.45% ( ±0.07−0.13%), which are comparable to that (0.49 ± 0.13%) of pure LiYbF_4_:0.5%Tm^3+^@LiYF_4_ core/shell NPs (Supplementary Table 5). We can estimate the expected partial QYs of the excitons in NP-sensitized PeQDs as being the product of the upconversion QYs of pure NPs by the efficiency of energy transfer and by the PLQYs of PeQDs; in this way, we attained 0.08, 0.38, and 0.28% for the chloride, bromide, and iodide perovskites, respectively, in good agreement with the experimental values mentioned above. This confirms the high photon conversion efficiency in NP-sensitized PeQDs. Note that higher upconversion QYs for CsPbX_3_ PeQDs could be achieved by improving both upconversion QYs of the NPs and PLQYs of PeQDs.

### Mechanistic investigation of the RETU process

**Fig. 4:**
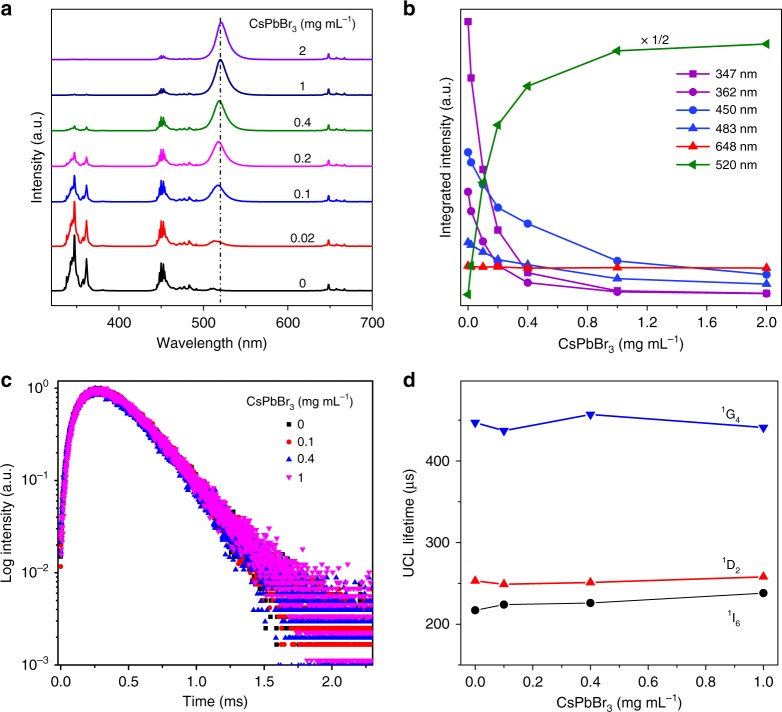
Mechanistic investigation of the radiative energy transfer upconversion (RETU) process in nanoparticle (NP)-sensitized CsPbX_3_ perovskite quantum dots (PeQDs). **a** CsPbBr_3_ concentration-dependent upconversion luminescence (UCL) spectra for LiYbF_4_:0.5%Tm^3+^@LiYF_4_ core/shell NP-sensitized CsPbBr_3_ PeQDs with NPs concentration of 1 mg mL^−1^ under 980 nm excitation at a power density of 50 W cm^−2^. **b** Integrated intensities of the Tm^3+^ emissions at 347, 362, 450, 483, and 648 nm and the CsPbBr_3_ emission at 520 nm vs. the CsPbBr_3_ concentration, as obtained from (**a**). **c** UCL decays from ^1^D_2_ of Tm^3+^ by monitoring the Tm^3+^ emission at 362 nm in NP-sensitized CsPbBr_3_ PeQDs with varying CsPbBr_3_ concentrations under 980 nm excitation. **d** UCL lifetimes of ^1^I_6_, ^1^D_2_, and ^1^G_4_ of Tm^3+^ in NP-sensitized CsPbBr_3_ PeQDs vs. the CsPbBr_3_ concentration

In view of the core/shell structure of the NPs that imposed a distance of 5.5 nm between the energy donor and acceptor, we deduced that the energy transfer from the NPs to PeQDs is dominated by a radiative reabsorption process instead of a non-radiative FRET process, because the core/shell structure is unfavorable for distance-dependent FRET^49,50^. To gain more insights into the energy transfer process, we investigated PeQDs concentration-dependent steady-state and transient UCL in NP-sensitized PeQDs. As shown in Fig. 4a, under 980 nm CW diode laser excitation, the CsPbBr_3_ emission at 520 nm increased gradually at the expense of the UV and blue emissions of Tm^3+^ with increasing CsPbBr_3_ concentration, as a result of energy transfer from the NPs to CsPbBr_3_ PeQDs. Coincidentally, the UV emission intensities of Tm^3+^ at 347 nm (^1^I_6_ → ^3^F_4_) and 362 nm (^1^D_2_ → ^3^H_6_) dropped much faster than the blue emissions at 450 nm (^1^D_2_ → ^3^F_4_) and 483 nm (^1^G_4_ → ^3^H_6_) with increasing CsPbBr_3_ concentration, whereas the red emissions at 648 nm (^1^G_4_ → ^3^F_4_) remained nearly unchanged (Fig. 4b). This can be attributed to the much larger absorbance of CsPbBr_3_ in UV than in blue and no absorption in red (Supplementary Fig. 9). One should keep in mind that the depopulation of ^1^D_2_ or ^1^G_4_ of Tm^3+^ through non-radiative FRET would lead to simultaneous quenching of all emissions from ^1^D_2_ or ^1^G_4_^32^. Thus, the observed distinct UCL evolutions between the emissions from identical ^1^D_2_ or ^1^G_4_ level reveal that the NPs-to-PeQDs energy transfer may be a radiative reabsorption process rather than a non-radiative FRET process. Such radiative energy transfer was further evidenced by the essentially unchanged UCL lifetimes of ^1^I_6_, ^1^D_2_, and ^1^G_4_ of Tm^3+^ in NP-sensitized CsPbBr_3_ PeQDs in comparison with those in NPs alone (Fig. 4c, d and Supplementary Fig. 10), because non-radiative FRET always results in a decrease in lifetime of energy donor by imposing additional relaxation channel on the donor^51^. The drastically distinct evolutions in PeQDs concentration-dependent UCL between the emissions from ^1^D_2_ or ^1^G_4_ level and the nearly unchanged UCL lifetimes of Tm^3+^ were also observed for PeQDs with different halide compositions (Supplementary Figs. 11–18), thus demonstrating that the upconverted exciton emission from the NP-sensitized CsPbX_3_ PeQDs is governed by a RETU process.

### Upconversion lifetime tuning in NP-sensitized CsPbX_3_ PeQDs

**Fig. 5:**
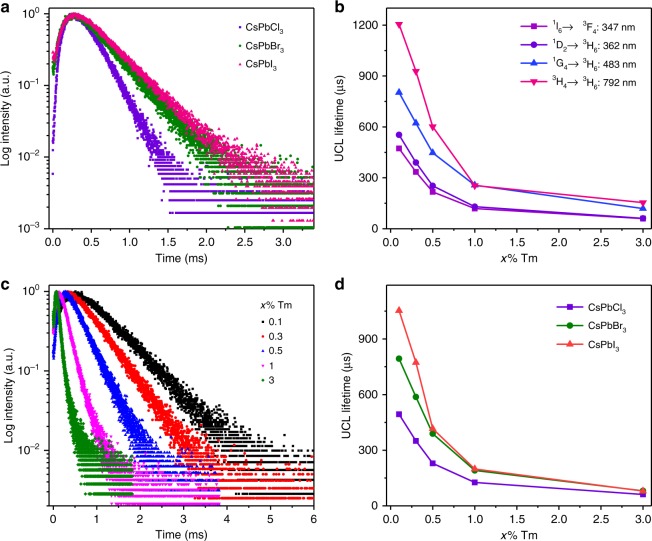
Upconversion lifetime tuning in nanoparticle (NP)-sensitized CsPbX_3_ perovskite quantum dots (PeQDs). **a** Upconversion luminescence (UCL) decays from the upconverted excitons in NP-sensitized CsPbCl_3_, CsPbBr_3_, and CsPbI_3_ PeQDs (0.5 mol% Tm^3+^) by monitoring their emissions at 410, 520, and 700 nm, respectively, under 980 nm excitation. **b** UCL lifetimes of ^1^I_6_, ^1^D_2_, ^1^G_4_, and ^3^H_4_ of Tm^3+^ in LiYbF_4_:x%Tm^3+^@LiYF_4_ core/shell NPs as a function of the Tm^3+^ concentration. **c** UCL decays from the upconverted excitons in NP-sensitized CsPbBr_3_ PeQDs with varying Tm^3+^ concentration in the NPs. **d** UCL lifetimes of CsPbCl_3_, CsPbBr_3_, and CsPbI_3_ PeQDs as a function of the Tm^3+^ concentration

More importantly, we discovered that the UCL lifetimes of exciton emissions in NP-sensitized PeQDs were abnormally much longer (hundreds of μs vs. ns) than their PL lifetimes in pure PeQDs. Figure 5a compares the UCL decays from the exciton emissions in NP-sensitized CsPbCl_3_, CsPbBr_3_, and CsPbI_3_ PeQDs, respectively, under 980 nm excitation. It was observed that all the decays displayed similar temporal profiles to that of Tm^3+^, namely, a rising edge in the initial stage followed by single-exponential decay, as a result of RETU process^52^. By single-exponential fitting to the decay curves, the UCL lifetimes of CsPbCl_3_, CsPbBr_3_, and CsPbI_3_ PeQDs were determined to be 229, 389, and 416 μs, respectively, in marked contrast to their original PL lifetimes of 1.8, 21.1, and 81.1 ns under UV excitation (Supplementary Table 2). It is worth mentioning that the exciton lifetimes of PeQDs were intrinsically unchanged, but were apparently lengthened due to the slow population of the PeQDs excited state from the long-lived Tm^3+^ excited state during the radiative energy transfer from the NPs to PeQDs^53,54^. Therefore, through lifetime tuning of Tm^3+^ in the NPs, the apparent UCL lifetimes of PeQDs can be modulated. By controlling the Tm^3+^ concentration from 3 mol% to 0.1 mol% in the NPs, the lifetimes of ^1^I_6_, ^1^D_2_, ^1^G_4_, and ^3^H_4_ of Tm^3+^ increased from 60, 61, 119, and 154 μs to 473, 553, 803, and 1205 μs, respectively (Fig. 5b and Supplementary Fig. 19). Accordingly, the UCL lifetimes of CsPbCl_3_, CsPbBr_3_, and CsPbI_3_ PeQDs sensitized by the NPs were tuned from 61, 81, and 80 μs to 494, 794, and 1053 μs, respectively (Fig. 5c, d). Similar strategy was employed for manipulating the UCL lifetime of exciton in hybrid CsPb(Cl/Br)_3_ and CsPb(Br/I)_3_ PeQDs (Supplementary Figs. 20–27 and Supplementary Table 6), thus validating the general lifetime tunability in various PeQDs. The ability of ultralong lifetime tuning (61 μs–1.053 ms) along with wide color gamut modulation (140% of the NTSC television color standard) via engineering the composition and concentration of PeQDs, offers us an unparalleled opportunity for constructing a huge library of discernable upconversion identities, which are of vital importance in optical encoding and multiplexing for applications in diverse areas such as complex data storage and multilevel anticounterfeiting^55,56^.

### Generality of the proposed RETU

**Fig. 6:**
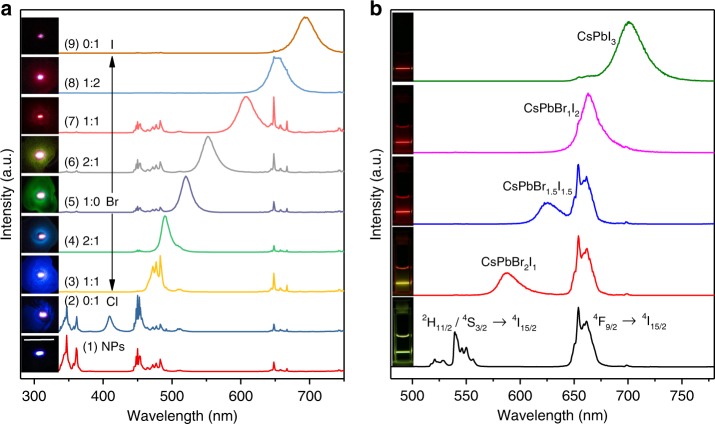
Generality of the proposed radiative energy transfer upconversion (RETU) for sensitizing photon upconversion in CsPbX_3_ perovskite quantum dots (PeQDs). **a** Upconversion luminescence (UCL) spectra for LiYbF_4_:0.5%Tm^3+^@LiYF_4_ core/shell nanoparticles (NPs) and the NP-sensitized CsPbX_3_ PeQDs (NPs: 1 mg mL^−1^; PeQDs: 2 mg mL^−1^) with varying halide compositions by casting them onto glass substrate under 980 nm CW diode laser excitation at a power density of 50 W cm^−2^. The insets show the corresponding UCL photographs for the NP-sensitized PeQDs, and the scale bar represents 1 cm. **b** UCL spectra for NaYF_4_:18%Yb^3+^, 2%Er^3+^ NPs (approximately 23 nm) and the NP-sensitized CsPbX_3_ PeQDs (NPs: 1 mg mL^−1^; PeQDs: 2 mg mL^−1^) with varying halide composition under 980 nm CW diode laser excitation at a power density of 20 W cm^−2^. The insets show the corresponding UCL photographs for the colloidal cyclohexane solution of the NP-sensitized PeQDs

Furthermore, we demonstrated that the NP-sensitized PeQDs can be fabricated as films by casting them onto glass substrate, from which intense and tunable upconversion emissions were realized under CW diode laser excitation (Fig. 6a). The proposed RETU nanosystem can also be extended to the sensitization of different kinds of quantum dots by employing different lanthanide-doped NPs as energy donors. For example, through sensitization by NaYF_4_:Yb^3+^, Er^3+^ NPs, tunable upconverted exciton emissions were detected in CsPbX_3_ PeQDs (Fig. 6b). Similarly, upconverted exciton emissions from CdSe and InP@ZnS quantum dots were explicitly observed through radiative energy transfer from LiYbF_4_:Tm^3+^@LiYF_4_ (Supplementary Figs. 28 and 29). These results show the generality of RETU for sensitizing photon upconversion in various quantum dots.

## Discussion

In contrast to multiphoton absorption, the proposed RETU in NP-sensitized CsPbX_3_ PeQDs is made much more efficient through the use of physically existing intermediate energy levels of lanthanide ions and thus can be triggered by using a low-cost NIR laser diode. In comparison with non-radiative FRET, the RETU nanosystem breaks the distance restriction on energy transfer efficiency, and therefore is much more robust and convenient through simple mixing of lanthanide-doped NPs and PeQDs. Most importantly, as a merit of RETU, the PL lifetime of the exciton in PeQDs inheriting from the excited-state lifetime of lanthanides can be lengthened tremendously from the original nanosecond scale to milliseconds. Such a unique PL lifetime of the exciton is beneficial for electron and hole separation in photovoltaics by distributing the large number of excitons in a much longer time window, and is also highly desirable for time-gated PL biosensing to eliminate the interference of short-lived background noise. Nevertheless, there remain many technical challenges to be overcome toward their practical applications, including the concerns about the stability and toxicity of PeQDs, as well as their upconversion efficiency. We envision that these concerns can be addressed by overall optimization involving the controlled synthesis and surface modification of the NP-sensitized PeQDs. For example, we can encapsulate lanthanide-doped NPs and PeQDs into SiO_2_ or polystyrene NPs to improve the chemical stability, and employ dye-sensitized lanthanide-doped NPs as an antenna to broaden the excitation wavelengths and boost the upconversion efficiency. Finally, it is urgent to develop lead-free PeQDs with desirable physicochemical properties for optoelectronics and photovoltaics.

To conclude, the optical properties observed in lanthanide-doped NP-sensitized CsPbX_3_ PeQDs are striking because they have presented the first panorama of photon upconversion with high efficiency and multicolor and lifetime tunability in PeQDs under low power density excitation. These findings offer a general approach for fine tuning NIR-triggered photon upconversion in PeQDs, which may open up a new avenue for the exploration of PeQDs toward versatile applications, such as ultrasensitive bioassay, high-resolution bioimaging, and full-spectrum conversion in solar cells.

## Methods

### Chemicals and materials

Pb(CH_3_COO)_2_·3H_2_O (99.99%), CH_3_COOLi·2H_2_O (99.99%), Cs_2_CO_3_ (99.99%), HBr (48%), HI (55.0–58.0%) were purchased from Aladdin (Shanghai, China). Oleic acid (OA, 90%), oleylamine (OAm, 90%), 1-octadecene (ODE, 90%), trioctylamine (98%), Yb(CH_3_COO)_3_·4H_2_O (99.999%), Tm(CH_3_COO)_3_·4H_2_O (99.99%), and Y(CH_3_COO)_3_·4H_2_O (99.99%) were purchased from Sigma-Aldrich (Shanghai, China). NH_4_F, HCl, cyclohexane, acetone, and ethanol were of analytical grade and purchased from Sinopharm Chemical Reagent Co. (Shanghai, China). All the chemical reagents were used as received without further purification.

### Synthesis of CsPbX_3_ PeQDs

Monodisperse CsPbX_3_ (X = Cl, Br, and I) PeQDs were synthesized through a modified hot-injection method by using HX as the halide source to precipitate the PeQDs^45^. As a result, the halide composition of the PeQDs can be precisely adjusted on demand by changing the ratio of HX during the synthesis. In a typical synthesis of CsPbBr_3_ PeQDs, 0.5 mmol of Pb(CH_3_COO)_3_·3H_2_O and 0.1 mmol of Cs_2_CO_3_ were mixed with 2 mL of OA, 2 mL of OAm, and 6 mL of ODE in a 50 mL three-neck round-bottom flask. The resulting mixture was heated to 120 °C under a N_2_ flow with constant stirring for 40 min to form a clear solution. The temperature was then raised up to 180 °C and stabilized for 10 min, and 1.5 mmol of HBr was quickly injected. After 20 s, the reaction mixture was cooled down to room temperature by ice-water bath. For the synthesis of CsPbCl_3_ and CsPbI_3_ PeQDs, 1.5 mmol of HCl and HI instead of HBr was injected, respectively, under otherwise identical conditions. For the synthesis of mixed-halide CsPb(Cl/Br)_3_ and CsPb(Br/I)_3_ PeQDs, 1.5 mmol of total amount of mixed HCl/HBr and HBr/HI was injected, respectively. The final halide composition of the PeQDs was designated by the ratio of HCl/HBr/HI used in the synthesis.

### Isolation and purification of CsPbX_3_ PeQDs

The crude solution was cooled down to room temperature with ice-water bath and the PeQDs were collected by centrifuging at 12,000 rpm for 5 min. The precipitate was then dispersed in 1 mL of cyclohexane and centrifuged again at 12,000 rpm for 5 min. After centrifugation, the supernatant was discarded and the PeQDs were redispersed in 30 mL of cyclohexane. Finally, 100 μL of OAm was added and ultrasonicated for 1 min to stabilize the PeQDs for long-term storage.

### Structural and optical characterization

Powder X-ray powder diffraction patterns of the samples were collected with an X-ray diffractometer (MiniFlex2, Rigaku) with Cu Kα1 radiation (*λ* = 0.154187 nm). Both the low- and high-resolution TEM measurements were performed by using a TECNAI G^2^ F20 TEM equipped with the energy dispersive X-ray spectrum. Optical absorption spectra of CsPbX_3_ PeQDs were collected with a Perkin-Elmer Lambda365 UV/Vis spectrometer in transmission mode. PL excitation and emission spectra and PL decays were recorded on the FLS980 spectrometer (Edinburgh) equipped with both continuous xenon (450 W) and pulsed flash lamps. UCL emission spectra were acquired under 980 nm excitation with a CW diode laser (2 W). UCL lifetimes were measured with a customized UV to mid-infrared steady-state and phosphorescence lifetime spectrometer (FSP920-C, Edinburgh) equipped with a digital oscilloscope (TDS3052B, Tektronix) and a tunable mid-band Optical Parametric Oscillator pulsed laser as the excitation source (410–2400 nm, 10 Hz, pulse width ≤ 5 ns, Vibrant 355II, OPOTEK). PL photographs of the NPs and PeQDs were taken by using a Canon 70D digital camera without using any filter. The absolute PLQYs of CsPbX_3_ PeQDs were measured by employing a standard barium sulfate coated integrating sphere (150 mm in diameter, Edinburgh) as the sample chamber that was mounted on the FLS920 spectrometer with the entry and output port of the sphere located in 90° geometry from each other in the plane of the spectrometer. A standard tungsten lamp was used to correct the optical response of the instrument. All the spectral data were recorded at room temperature and corrected for the spectral response of both the spectrometer and the integrating sphere.

### Data availability

The authors declare that all data supporting the findings of this study are available within the paper and its supplementary information files.

## Electronic supplementary material

Supplementary Information
