# Supplementary material for: Near-infrared-triggered photon upconversion tuning in all-inorganic cesium lead halide perovskite quantum dots
Source: Nat Commun. 2018 Aug 27;9:3462. doi: 10.1038/s41467-018-05947-2 (PMC6110834; doi:10.1038/s41467-018-05947-2)
Supplement: Supplementary file 1 — Supplementary Information [file 41467_2018_5947_MOESM1_ESM.pdf]

Near-infrared triggered photon upconversion tuning in all-inorganic cesium lead halide perovskite quantum dots

Zheng et al.

Supplementary Note 1: Synthesis of $LiYbF_4$:Tm^{3+} NPs. Monodisperse $LiYbF_4$:Tm^{3+} nanoparticles (NPs) were synthesized through a high-temperature co-precipitation method as we previously reported (Supplementary Ref. 1). In a typical synthesis of $LiYbF_4$:0.5%Tm^{3+} NPs, 1 mmol of $CH_3COOLi·2H_2O$, 0.995 mmol of $Yb(CH_3COO)_3·4H_2O$ and 0.005 mmol of $Tm(CH_3COO)_3·4H_2O$ were mixed with 8 mL of oleic acid (OA) and 12 mL of trioctylamine (TOA) in a 100 mL three-necked round-bottom flask. The resulting mixture was heated to 160 °C under a N_2 flow with constant stirring for 30 min to form a clear solution, and then cooled down to room temperature. Thereafter, 10 mL of methanol solution containing 4 mmol of NH_4F was added and the solution was stirred at 60 °C for 30 min to remove methanol. After methanol was evaporated, the resulting solution was heated to 320 °C under a N_2 flow with vigorous stirring for 60 min, and then cooled down to room temperature. The obtained NPs were precipitated by addition of 30 mL of ethanol, collected by centrifugation, washed with ethanol several times, and finally redispersed in cyclohexane. To control the doping concentration of Tm^{3+} in the resulting NPs, different amounts of $Yb(CH_3COO)_3·4H_2O$ and $Tm(CH_3COO)_3·4H_2O$ were used in the synthesis under otherwise identical conditions.

Synthesis of $LiYbF_4$:Tm^{3+}@$LiYF_4$ core/shell NPs. $LiYbF_4$:Tm^{3+}@$LiYF_4$ core/shell NPs were synthesized through a seed-mediated growth strategy by using $LiYbF_4$:Tm^{3+} core-only NPs as the seed. In a typical synthesis, 0.5 mmol of $CH_3COOLi·2H_2O$ and 0.5 mmol of $Y(CH_3COO)_3·4H_2O$ were added to a 100 mL three-necked round-bottom flask containing 8 mL of OA and 12 mL of TOA. The mixed solution was then heated to 160 °C under a N_2 flow with constant stirring for 30 min to form a clear solution. After cooling down to 80 °C, 0.5 mmol of $LiYbF_4$:Tm^{3+} core-only NPs in 10 mL of cyclohexane was added and maintained at 80 °C for 30 min to remove cyclohexane. After the removal of cyclohexane, 10 mL of methanol solution containing 2 mmol of NH_4F was added and stirred at 60 °C for another 30 min to remove methanol. After methanol was evaporated, the solution was heated to 320 °C under a N_2 flow with vigorous stirring for 60 min, and then cooled down to room temperature. The resulting core/shell NPs were precipitated by addition of 30 mL of ethanol, collected by centrifugation, washed with ethanol several times, and finally redispersed in cyclohexane.

Supplementary Note 2: Determination of the energy transfer efficiency in NPs-sensitized $CsPbX_3$ PeQDs. We defined the efficiency of energy transfer (η^{ETE}) from $LiYbF_4$:0.5%Tm^{3+}@$LiYF_4$ NPs to $CsPbX_3$ perovskite quantum dots (PeQDs) as the ratio of the number of emitted photons from Tm^{3+} that are absorbed by PeQDs (n^{x}_{abs}) in NPs-sensitized PeQDs to the number of emitted photons from Tm^{3+} (n^{Tm}_{em}) with frequencies above the band edge of PeQDs in pure $LiYbF_4$:0.5%Tm^{3+}@$LiYF_4$ core/shell NPs upon 980 nm continuous-wave (CW) diode laser excitation under identical conditions. Therefore, based on the upconversion luminescence (UCL) spectra, the energy transfer efficiency can be calculated by

$$\eta^{ETE} = \frac{n^{x}_{abs}}{n^{Tm}_{em}} = \frac{I^{Tm}_{0} - I^{Tm}_{x}}{I^{Tm}_{0}}$$

where I^{Tm}_{0} and I^{Tm}_{x} represent the integrated intensity of the UCL of Tm^{3+} from the NPs with frequencies above the band edge of $CsPbX_3$ in the absence and presence of $CsPbX_3$ PeQDs, respectively. Note that the ultraviolet (UV) emissions from Tm^{3+} at 289 nm in pure NPs were also integrated for calculation, though that they were totally quenched in NPs-sensitized PeQDs.

Supplementary Note 3: Determination of the absolute UCQYs. The absolute upconversion quantum yields (QYs) were measured on a customized absolute upconversion QY (UCQY) measurement system combined with a fiber optic spectrometer (QE65pro, Ocean Optics), a standard barium sulfate coated integrating sphere (150 mm in diameter, Edinburgh), a 980 nm CW diode laser (MDL-III-980-2W, Changchun New Industries Optoelectronics Tech Co., Ltd.) as the excitation source, and a neutral density filter to attenuate the excitation light. The measurement was conducted deliberately according to the protocols reported by van Veggel and U. R. Genger *et al.* (Supplementary Ref. 2 and 3). The integrating sphere was mounted on the optical platform with the entry and output ports of the sphere located in 90° geometry from each other. All the solution samples of the NPs and PeQDs were mounted in a quartz cuvette located in the center of the integrating sphere. Pure cyclohexane solution for reference were mounted in another quartz cuvette in the integrating sphere with a distance of 1 cm away from the samples. Samples were excited with a 980 nm CW diode laser. An optical lens was used to collimate the laser beam and direct on the sample with a foucs of 1 mm^2 and a laser powermeter (Model 1918-C, Newport) was applied to

measure the excitation power on the sample. The absorption of the samples at 980 nm was measured by changing the position of the samples and the reference, and calculated by their difference of the corrected excitation spectra. Baffles were employed on both sides of the sample holder to ensure that no scattered excitation or emission light would be collected before scattering off the inside of the sphere. All the spectroscopic data collected were corrected for the spectral response of both the spectrometer and the integrating sphere. The response of the detection systems in photon flux (integrating sphere, monochromators, and detectors) was determined using a calibrated tungsten lamp (100 W, Edinburgh). The normalization curves were then applied to all measured spectra. The UCQYs were then calculated by (Supplementary Ref. 2)

$$QY = \frac{N^{\mathrm{e}}}{N^{\mathrm{a}}} = \frac{L^{\mathrm{S}}}{E^{\mathrm{R}} - E^{\mathrm{S}}}$$

where N^{e} and N^{a} are the photons emitted and absorbed, respectively; L^{S} is the emission intensity, E^{R} and E^{S} are the intensities of the excitation light in the presence of the reference and the samples, respectively. The upconversion emission peaks in the spectral region of 330-740 nm were integrated for the QY determination. All the UCQYs for each sample were measured independently for three times under identical conditions to yield the average value and standard deviation.

Supplementary Table 1. Photoluminescence (PL) parameters of $CsPbX_3$ PeQDs with varying halide compositions upon UV excitation at 360 nm: emission peak wavelength, full-width at half maximum (FWHM), and absolute PLQY. The absolute PLQYs for each sample were measured independently for three times under identical conditions to yield the average value and standard deviation.

Sample	Emission Peak (nm)	FWHM (nm)	PLQY (%)
$CsPbCl_3$	410	11.7	26.0 ± 0.3
$CsPbCl_2Br_1$	444	15.5	35.6 ± 0.4
$CsPbCl_{1.5}Br_{1.5}$	464	16.3	61.8 ± 0.4
$CsPbCl_1Br_2$	481	17.9	61.4 ± 0.8
$CsPbBr_3$	520	20.3	79.8 ± 0.9
$CsPbBr_2I_1$	587	28.2	71.2 ± 0.5
$CsPbBr_{1.5}I_{1.5}$	631	29.4	62.7 ± 0.2
$CsPbBr_1I_2$	660	29.9	58.5 ± 0.7
$CsPbI_3$	700	37.0	57.5 ± 0.4

Supplementary Table 2. Effective PL lifetimes of $CsPbX_3$ PeQDs with varying halide compositions upon excitation with a 397 nm picosecond pulsed laser. τ^{eff} was calculated by

$$\tau^{eff} = \frac{1}{I^{max}} \int_0^{\infty} I(t) dt,$$

where $I(t)$ denotes the PL intensity as a function of time t, and I^{max} is the maximum PL intensity. The errors of the lifetimes are smaller than 5%.

Samples	τ^{eff} (ns)	Samples	τ^{eff} (ns)	Samples	τ^{eff} (ns)
$CsPbCl_3$	1.8	$CsPbCl_1Br_2$	8.2	$CsPbBr_{1.5}I_{1.5}$	66.2
$CsPbCl_2Br_1$	3.8	$CsPbBr_3$	21.1	$CsPbBr_1I_2$	75.0
$CsPbCl_{1.5}Br_{1.5}$	8.9	$CsPbBr_2I_1$	41.1	$CsPbI_3$	81.1

Supplementary Table 3. Chromaticity coordinates (CIE, 1931) for the UCL from pure $LiYbF_4$:0.5%Tm^{3+}@$LiYF_4$ core/shell NPs and NPs-sensitized $CsPbX_3$ PeQDs with varying halide compositions under 980 nm CW diode laser excitation at a power density of 50 W cm^{-2}. Chromaticity coordinates for the blue emission from $CsPbCl_{1.5}Br_{1.5}$, green emission from $CsPbBr_3$, and red emission from $CsPbBr_{1.5}I_{1.5}$ under 360 nm excitation were also outlined for comparison.

Sample	*x*	*y*	Sample	*x*	*y*
pure NPs	0.154	0.071	NPs/$CsPbBr_{1.5}I_{1.5}$	0.677	0.320
NPs/$CsPbCl_3$	0.155	0.063	NPs/$CsPbBr_1I_2$	0.716	0.281
NPs/$CsPbCl_{1.5}Br_{1.5}$	0.129	0.091	NPs/$CsPbI_3$	0.711	0.273
NPs/$CsPbCl_1Br_2$	0.076	0.341	$CsPbBr_3$ (green, PL)	0.112	0.787
NPs/$CsPbBr_3$	0.135	0.734	$CsPbCl_{1.5}Br_{1.5}$ (blue, PL)	0.134	0.053
NPs/$CsPbBr_2I_1$	0.507	0.485	$CsPbBr_{1.5}I_{1.5}$ (red, PL)	0.689	0.311

Supplementary Table 4. Calculated energy transfer efficiency (η^{ETE}) in NPs-sensitized $CsPbX_3$ PeQDs with varying halide compositions under 980 nm CW diode laser excitation at a power density of 50 W cm^{-2}.

Composition	η^{ETE}	Composition	η^{ETE}
$CsPbCl_3$	65.5%	$CsPbBr_2I_1$	98.2%
$CsPbCl_{1.5}Br_{1.5}$	94.4%	$CsPbBr_{1.5}I_{1.5}$	99.3%
$CsPbCl_1Br_2$	96.8%	$CsPbBr_1I_2$	99.8%
$CsPbBr_3$	96.6%	$CsPbI_3$	99.9%

Supplementary Table 5. UCQYs for pure $LiYbF_4$:0.5%Tm^{3+}@$LiYF_4$ core/shell NPs and NPs-sensitized $CsPbX_3$ PeQDs with varying halide compositions under 980 nm CW diode laser excitation at a power density of 100 W cm^{-2}. For comparison, UCQYs of the UV, blue, and red emissions of Tm^{3+} from the NPs and the upconverted exciton emissions from PeQDs were calculated, respectively. All UCQYs were measured independently for three times under identical conditions to yield the average value and standard deviation.

Sample	Emission group	Integrated spectral region (nm)	UCQY (%)
Pure NPs	UV	330-400	0.15 ± 0.04
	blue	430-530	0.29 ± 0.07
	red	630-700	0.05 ± 0.01
	overall	330-700	0.49 ± 0.13
NPs/$CsPbCl_3$	UV (Tm^{3+})	330-380	0.08 ± 0.02
	violet (exciton)	380-435	0.06 ± 0.01
	blue (Tm^{3+})	435-530	0.26 ± 0.03
	red (Tm^{3+})	630-700	0.05 ± 0.01
	overall	330-700	0.44 ± 0.07
NPs/$CsPbCl_{1.5}Br_{1.5}$	blue (exciton)	400-530	0.35 ± 0.07
	red (Tm^{3+})	630-700	0.06 ± 0.01
	overall	400-700	0.41 ± 0.08
NPs/$CsPbCl_1Br_2$	blue (exciton)	420-530	0.40 ± 0.10
	red (Tm^{3+})	630-700	0.05 ± 0.01
	overall	420-700	0.45 ± 0.11
NPs/$CsPbBr_3$	green (exciton)	430-580	0.39 ± 0.08
	red (Tm^{3+})	630-700	0.06 ± 0.02
	overall	430-700	0.45 ± 0.10
NPs/$CsPbBr_2I_1$	yellow (exciton)	520-630	0.39 ± 0.11
	red (Tm^{3+})	630-700	0.04 ± 0.02
	overall	520-700	0.43 ± 0.13
NPs/$CsPbBr_{1.5}I_{1.5}$	red (exciton, overall)	560-720	0.33 ± 0.06
NPs/$CsPbBr_1I_2$	red (exciton, overall)	570-720	0.33 ± 0.11
NPs/$CsPbI_3$	red (exciton, overall)	630-740	0.36 ± 0.09

Supplementary Table 6. UCL lifetimes of $^1I_6$, $^1D_2$, $^1G_4$, and $^3H_4$ of Tm^{3+} in pure $LiYbF_4$:x%Tm^{3+}@$LiYF_4$ core/shell NPs and UCL lifetimes of the upconverted excitons from NPs-sensitized $CsPbX_3$ PeQDs with varying halide compositions of PeQDs and Tm^{3+} concentrations of the NPs. The UCL lifetimes were obtained by single-exponential fitting to the decay curves. For non-exponential decay, the effective UCL lifetime was calculated by

$$\tau^{\mathrm{eff}} = \frac{1}{I^{\max}} \int_0^\infty I(t)dt$$

where $I(t)$ denotes the UCL intensity as a function of time t, and $I^{\max}$ represents the maximum UCL intensity. The errors of all lifetimes are smaller than 5%.

Transition	Emission (nm)	τ (μs) / x% Tm^{3+}				
		0.1	0.3	0.5	1	3
Tm^{3+}: $^1I_6 \rightarrow {}^3F_4$	347	473	335	217	119	60
Tm^{3+}: $^1D_2 \rightarrow {}^3H_6$	362	553	390	253	130	61
Tm^{3+}: $^1G_4 \rightarrow {}^3H_6$	483	803	622	447	257	119
Tm^{3+}: $^3H_4 \rightarrow {}^3H_6$	792	1205	928	601	255	154
$CsPbCl_3$	410	494	350	229	126	61
$CsPbCl_{1.5}Br_{1.5}$	467	978	719	398	196	79
$CsPbCl_1Br_2$	489	775	578	392	196	81
$CsPbBr_3$	520	794	587	389	191	81
$CsPbBr_2I_1$	580	742	618	437	299	198
$CsPbBr_{1.5}I_{1.5}$	630	840	656	443	226	100
$CsPbBr_1I_2$	657	727	572	438	282	243
$CsPbI_3$	700	1053	773	416	199	80

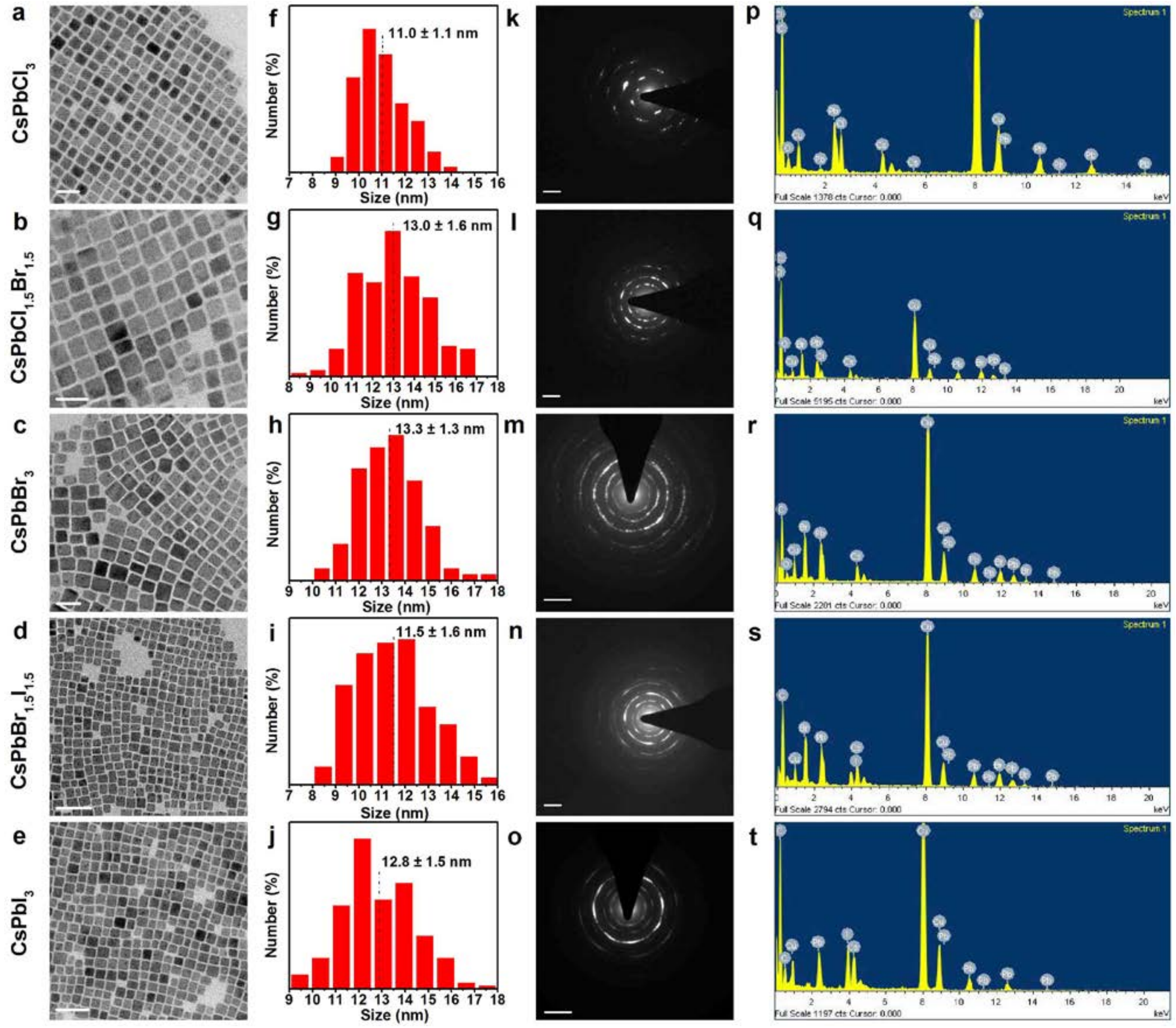

Supplementary Figure 1. **a-e** Transmission electron microscopy (TEM) images, **f-j** size distributions, **k-o** selected area electron diffraction (SAED) patterns, and **p-t** energy dispersive X-ray spectra of $CsPbX_3$ PeQDs with varying halide compositions. The scale bars in (**a-c**) and (**d**, **e**) represent 20 nm and 50 nm, respectively, and the scale bars in (**k-o**) are 2 nm^{-1}. TEM images showed that all PeQDs were roughly cubic with average lengths of 11.0-13.3 nm. The strong SAED ring patterns verified high crystallinity of the PeQDs. Energy dispersive X-ray spectra confirmed the elements of Cs, Pb, and X (X = Cl, Br, and I) in $CsPbX_3$ PeQDs. The size distributions were obtained by randomly calculating 200 particles in the TEM images.

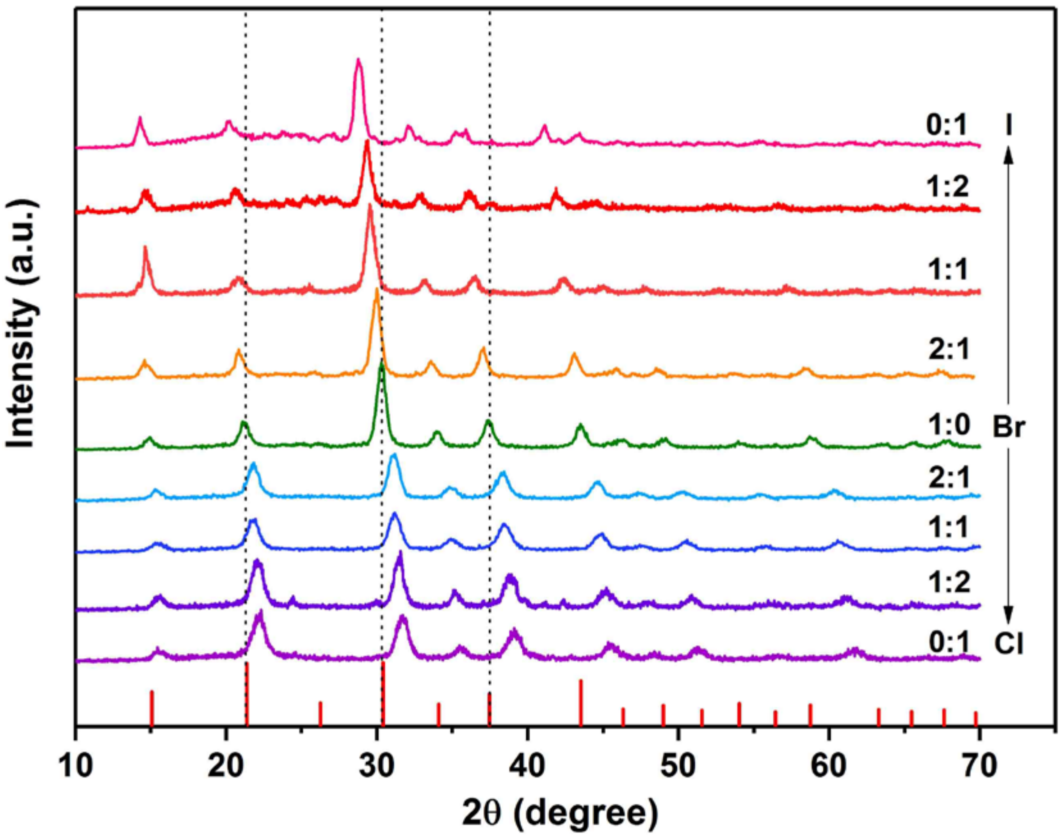

Supplementary Figure 2. X-ray powder diffraction (XRD) patterns of $CsPbX_3$ PeQDs with varying halide compositions. All the diffraction peaks matched well with the standard pattern of cubic $CsPbBr_3$ (JCPDS No. 75-0412), indicating pure phase and high crystallinity of the resulting PeQDs. A shift in the diffraction peaks toward lower angle was explicitly observed when the halide composition changed from Cl^- to Br^- and I^-, which can be ascribed to lattice expansion of PeQDs arising from the increased ionic radius of halide from Cl^- to I^-.

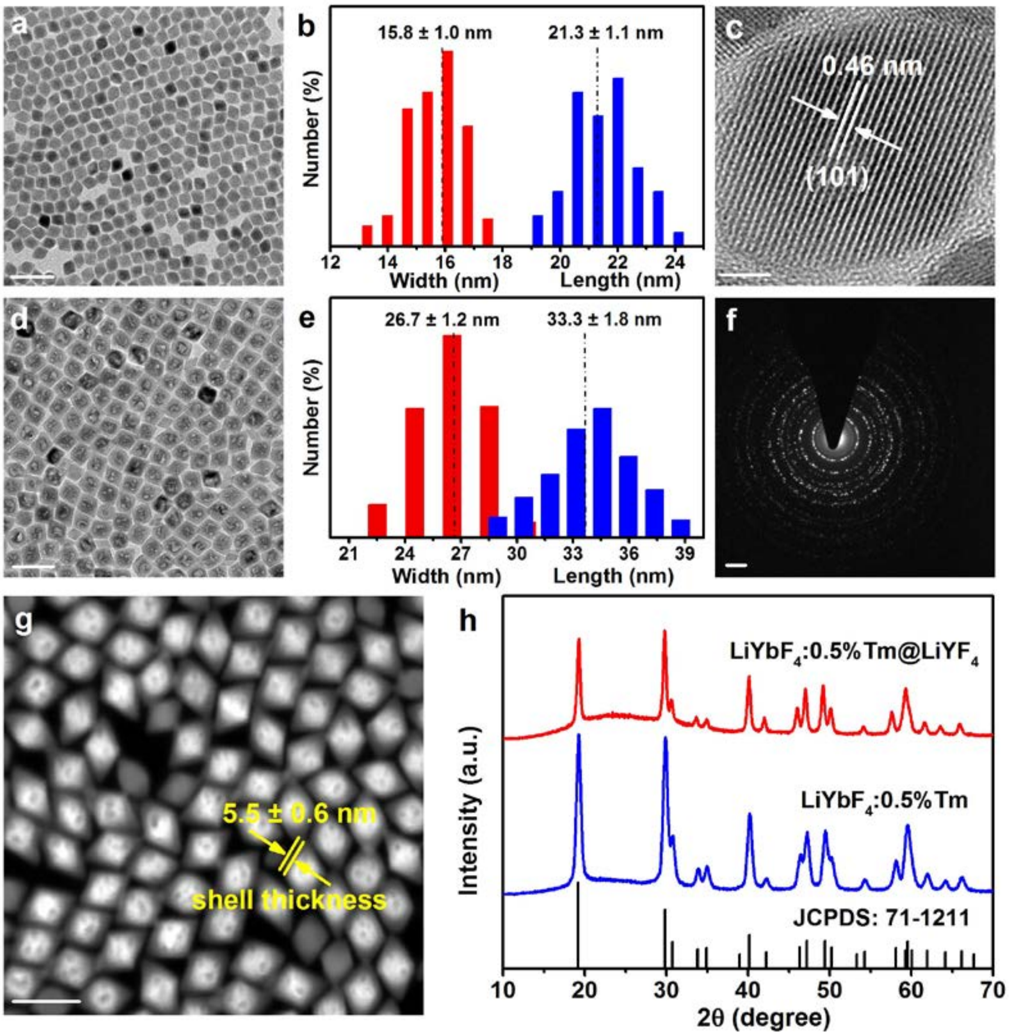

Supplementary Figure 3. **a** TEM image and **b** size distribution of $LiYbF_4$:0.5%Tm^{3+} core-only NPs. **c** High-resolution TEM image, **d** TEM image, **e** size distribution, **f** SAED pattern, and **g** high-angle annular dark-field scanning TEM (HAADF-STEM) image of $LiYbF_4$:0.5%Tm^{3+}@$LiYF_4$ core/shell NPs. The scale bars in (**a**, **c**, **d**, **f**, **g**) represent 50 nm, 5 nm, 50 nm, 2 nm^{-1}, and 50 nm, respectively. **h** XRD patterns of $LiYbF_4$:0.5%Tm^{3+} core-only and $LiYbF_4$:0.5%Tm^{3+}@$LiYF_4$ core/shell NPs. All diffraction peaks matched well with the standard pattern of tetragonal $LiYbF_4$ (JCPDS No. 71-1211), indicating pure phase and high crystallinity of the NPs. TEM images showed that both core-only and core/shell NPs were rhombohedral with their mean sizes of (15.8 ± 1.0) × (21.3 ± 1.3) nm and (26.7 ± 1.2) × (33.3 ± 1.8) nm, respectively. HAADF-STEM image showed a discernible contrasted core/shell structure with a shell thickness of 5.5 ± 0.6 nm, where the brighter regions correspond to the heavier Yb^{3+} ions in the cores, and the darker regions correspond to the lighter Y^{3+} ions in the shells. The high crystallinity of the NPs was confirmed by the intense SAED ring patterns and the high-resolution TEM image which exhibited clear lattice fringes with an observed d spacing of 0.46 nm for (101) plane of tetragonal $LiYbF_4$.

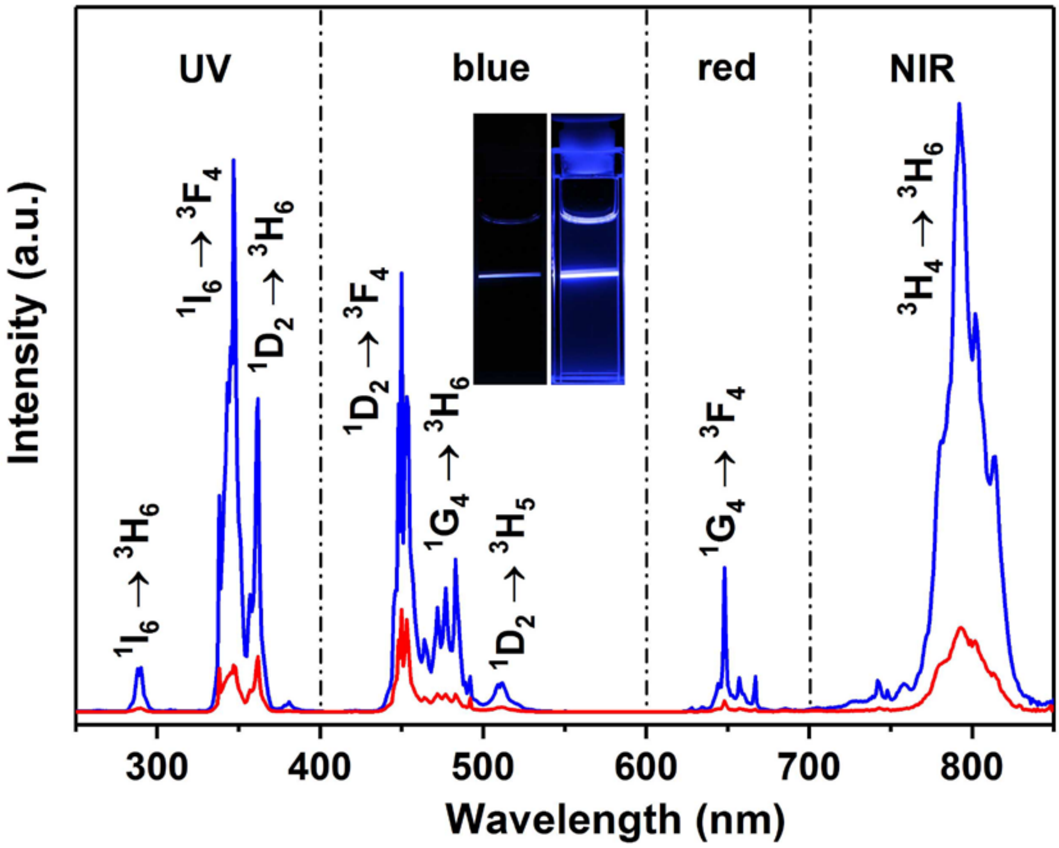

Supplementary Figure 4. UCL spectra of $LiYbF_4$:0.5%Tm^{3+} core-only (red) and $LiYbF_4$:0.5%Tm^{3+}@$LiYF_4$ core/shell (blue) NPs under 980 nm excitation with a CW diode laser at a power density of 50 W cm^{-2}. The insets show the corresponding UCL photographs for the colloidal cyclohexane solution of the NPs (~1 mg mL^{-1}) under 980 nm diode laser irradiation. Through surface passivation by growing an inert $LiYF_4$ shell, the integrated UCL intensity of the core/shell NPs was enhanced by a factor of 5.9 relative to their core-only counterparts, due to the decreased surface quenching effect.

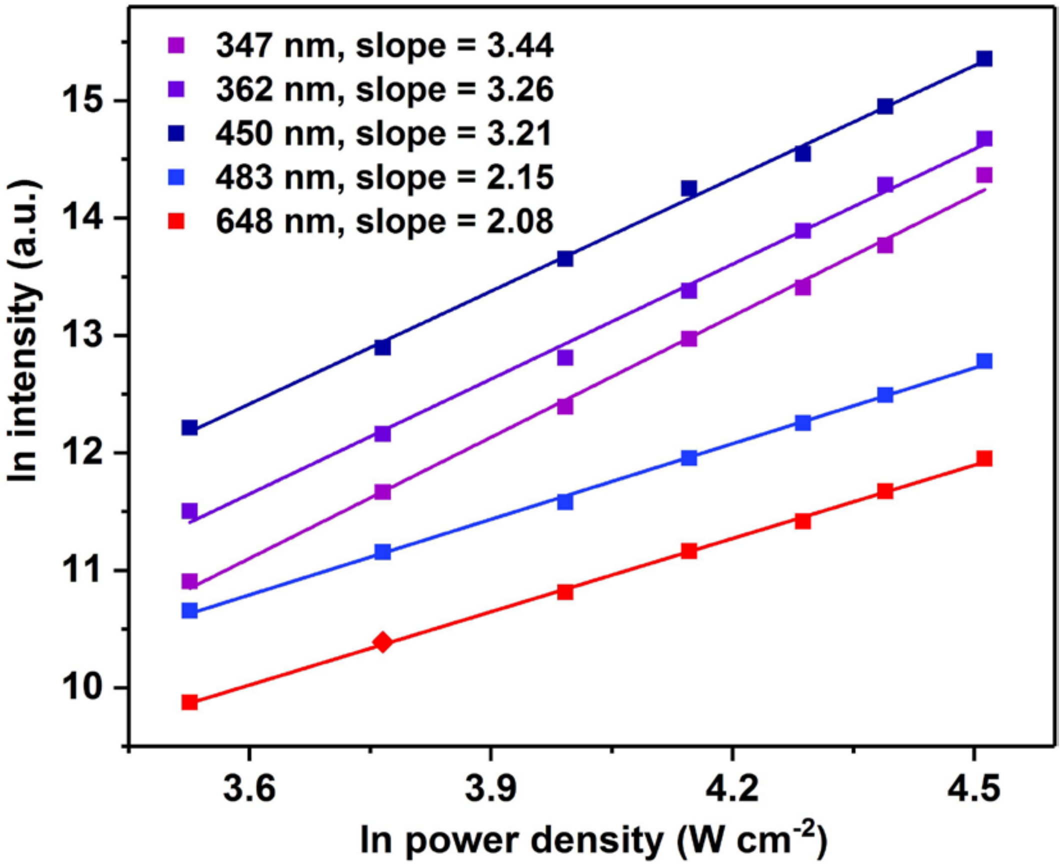

Supplementary Figure 5. Power density dependence of the Tm^{3+} emissions at 347 nm ($^1I_6 \rightarrow ^3F_4$), 362 nm ($^1D_2 \rightarrow ^3H_6$), 450 nm ($^1D_2 \rightarrow ^3F_4$), 483 nm ($^1G_4 \rightarrow ^3H_6$), and 648 nm ($^1G_4 \rightarrow ^3F_4$) in $LiYbF_4$:0.5%Tm^{3+}@$LiYF_4$ core/shell NPs, indicating five-, four-, and three-photon upconversion processes for the emissions from $^1I_6$, $^1D_2$, and $^1G_4$ level of Tm^{3+}, respectively. The deviation from the theoretically predicted photon number for the population of $^1I_6$ (n = 5), $^1D_2$ (n = 4) and $^1G_4$ (n = 3) of Tm^{3+} is most probably due to the upconversion saturation caused by the middle-to-high pump power density regime we employed.

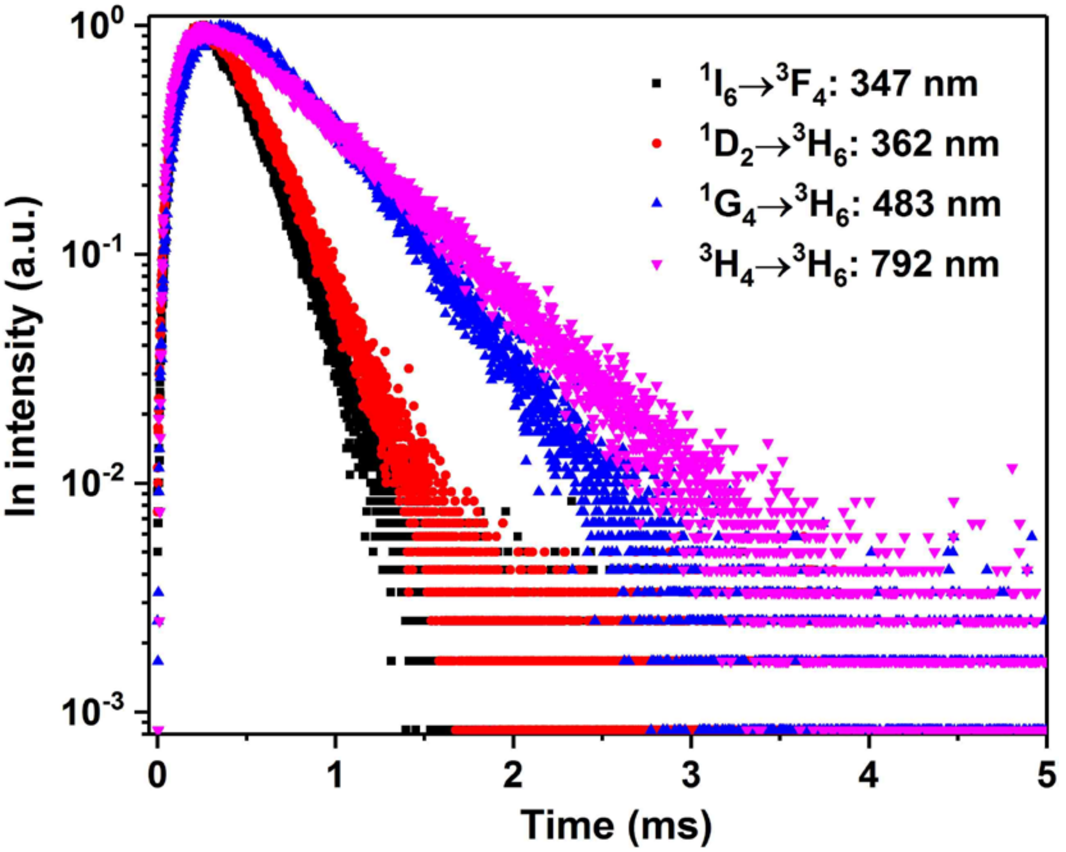

Supplementary Figure 6. UCL decays from $^1I_6$, $^1D_2$, $^1G_4$, and $^3H_4$ of Tm^{3+} by monitoring the Tm^{3+} emissions at 347, 362, 483, and 792 nm, respectively, in $LiYbF_4$:0.5%Tm^{3+}@$LiYF_4$ core/shell NPs under 980 nm excitation. All UCL decays displayed a rising edge in the initial stage, as a result of the slow population process of the emitting energy levels of Tm^{3+} through energy transfer from Yb^{3+}. By single-exponential fitting to the decay curves, the UCL lifetimes of $^1I_6$, $^1D_2$, $^1G_4$, and $^3H_4$ of Tm^{3+} were determined to be 217, 253, 447, and 601 μs, respectively.

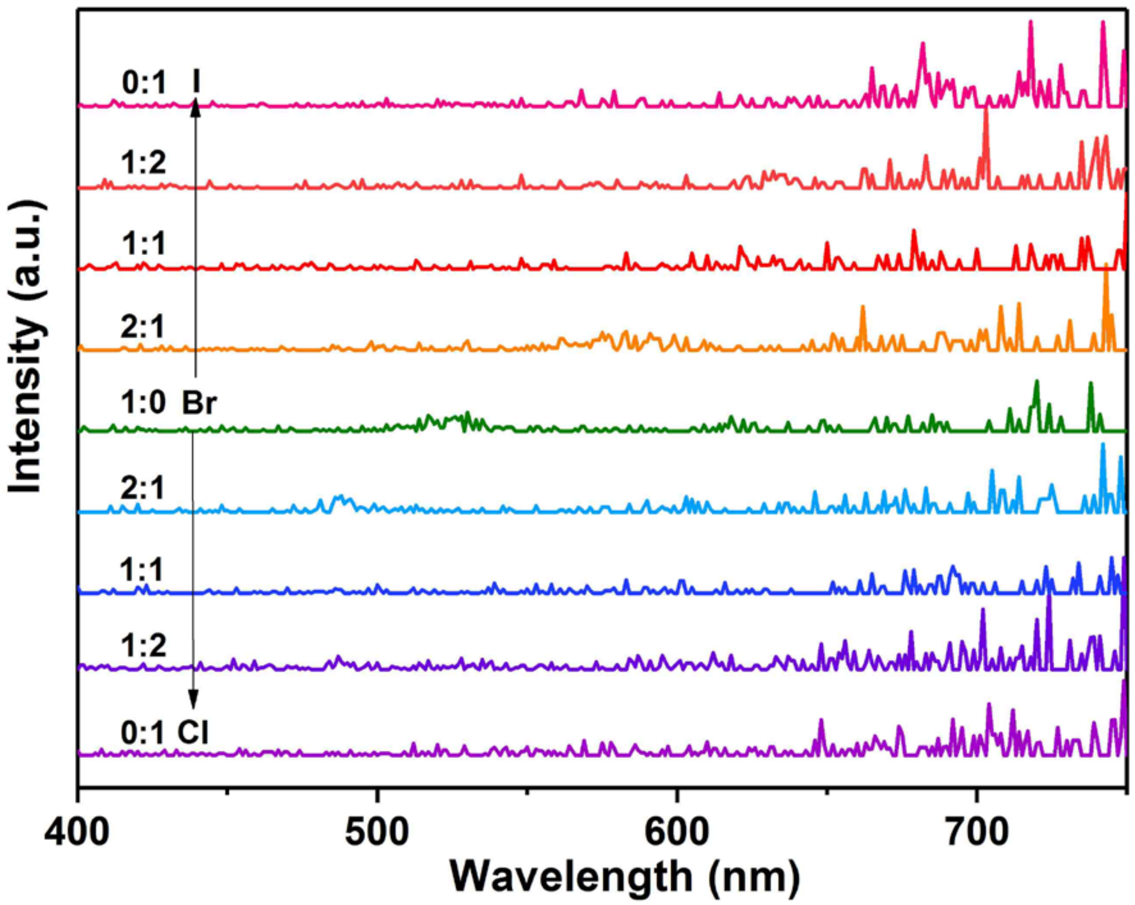

Supplementary Figure 7. UCL spectra of $CsPbX_3$ PeQDs with varying halide compositions under 980 nm excitation with a CW diode laser at a power density of 50 W cm^{-2}, showing that CW laser cannot trigger photon upconversion in pure PeQDs.

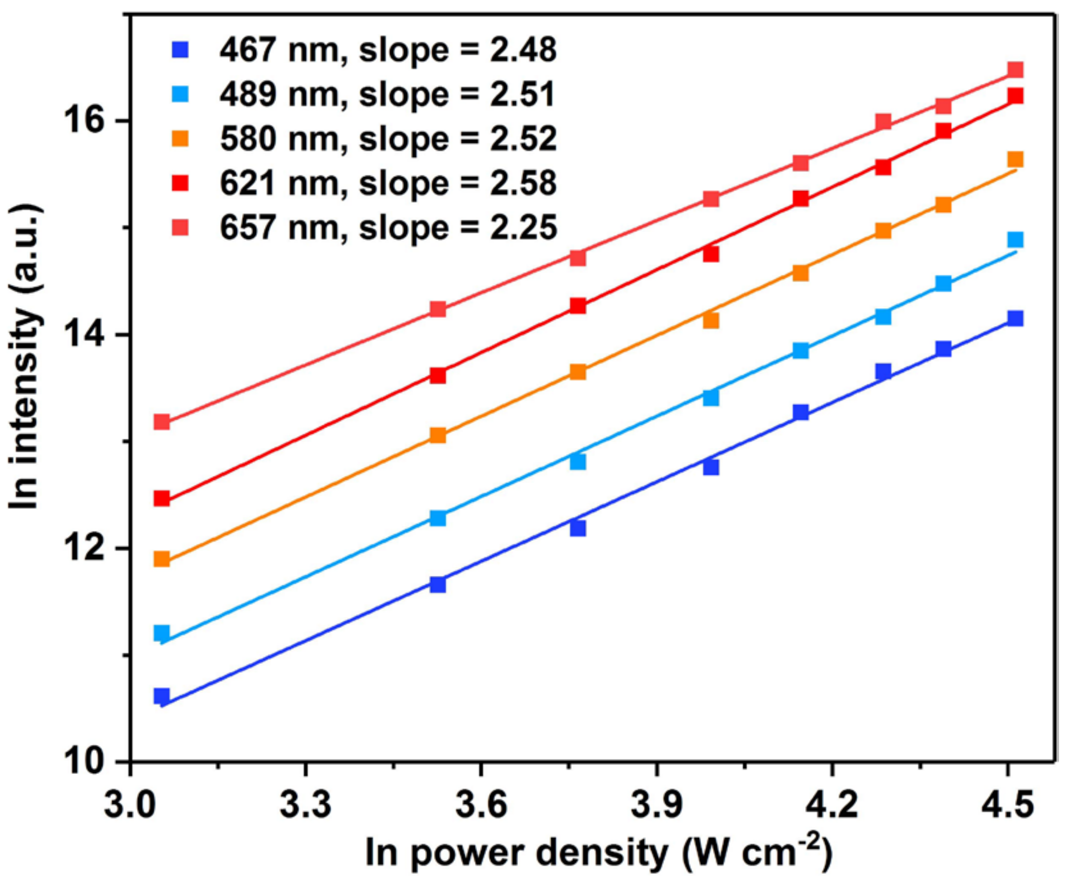

Supplementary Figure 8. Power density dependence of the upconverted exciton emissions from $CsPbCl_{1.5}Br_{1.5}$ at 467 nm, $CsPbCl_1Br_2$ at 489 nm, $CsPbBr_2I_1$ at 580 nm, $CsPbBr_{1.5}I_{1.5}$ at 621 nm, and $CsPbBr_1I_2$ at 657 nm in NPs-sensitized PeQDs under 980 nm CW diode laser excitation, showing a three-photon upconversion process for all emissions.

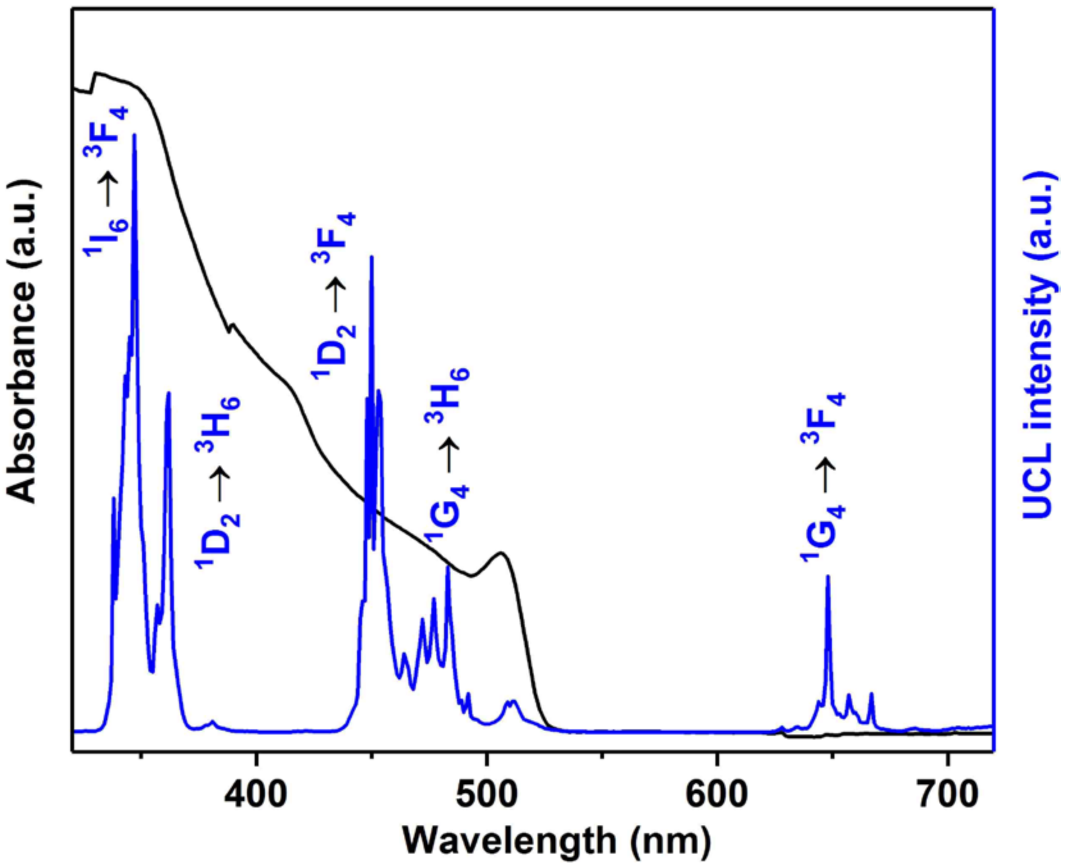

Supplementary Figure 9. Comparison of the absorption spectrum of $CsPbBr_3$ PeQDs (black) and the UCL spectrum of $LiYbF_4$:0.5%Tm^{3+}@$LiYF_4$ core/shell NPs (blue) under 980 nm CW diode laser excitation at a power density of 50 W cm^{-2}.

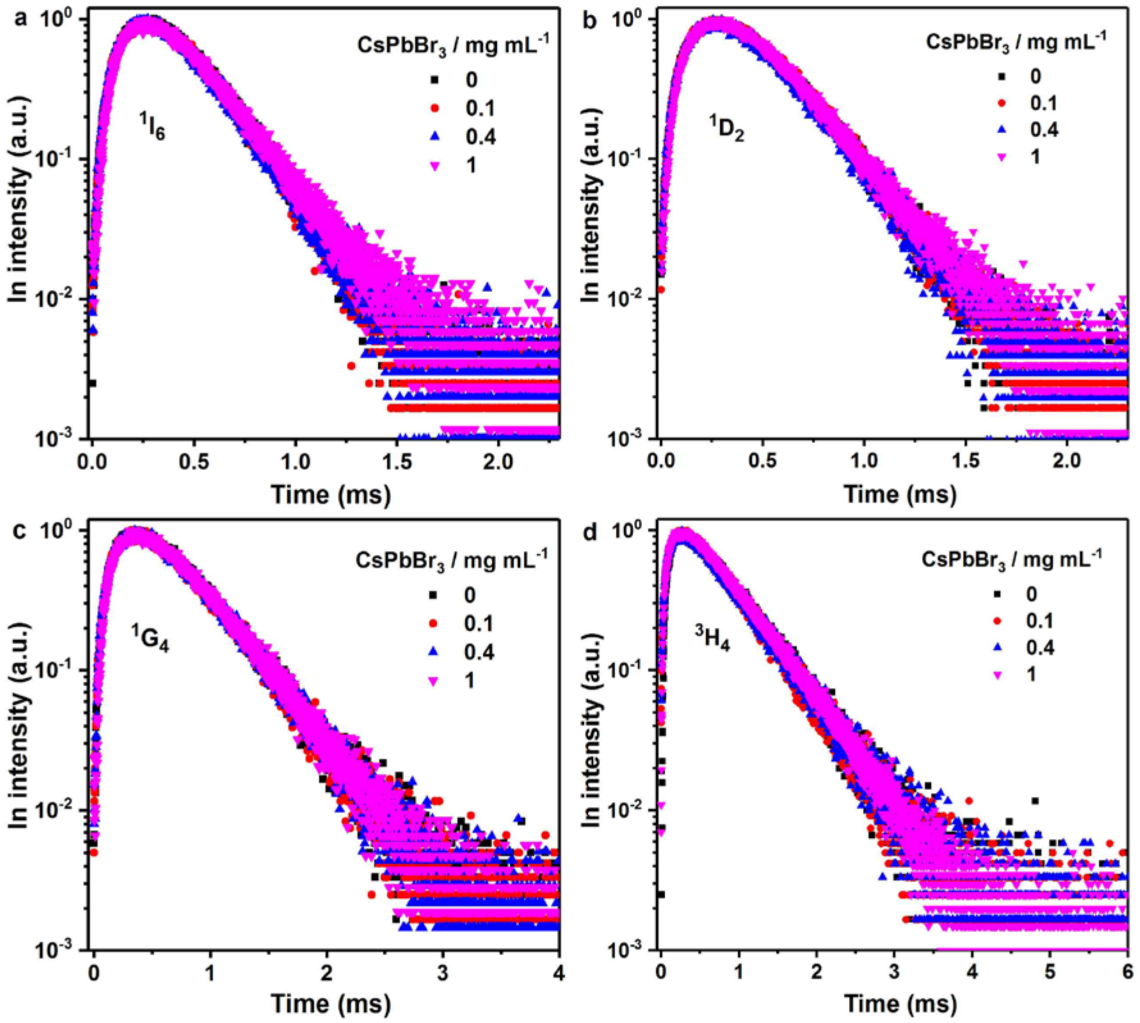

Supplementary Figure 10. $CsPbBr_3$ concentration-dependent UCL decays from **a** $^1I_6$, **b** $^1D_2$, **c** $^1G_4$, and **d** $^3H_4$ of Tm^{3+} by monitoring the Tm^{3+} emissions at 347, 362, 483, and 792 nm, respectively, in NPs-sensitized $CsPbBr_3$ PeQDs with NPs concentration of 1 mg mL^{-1} under 980 nm excitation. All UCL decays are nearly identical for each level of Tm^{3+} and are independent of the concentration and absorption of $CsPbBr_3$ PeQDs, thus providing a solid evidence for the radiative energy transfer from the NPs to $CsPbBr_3$ PeQDs.

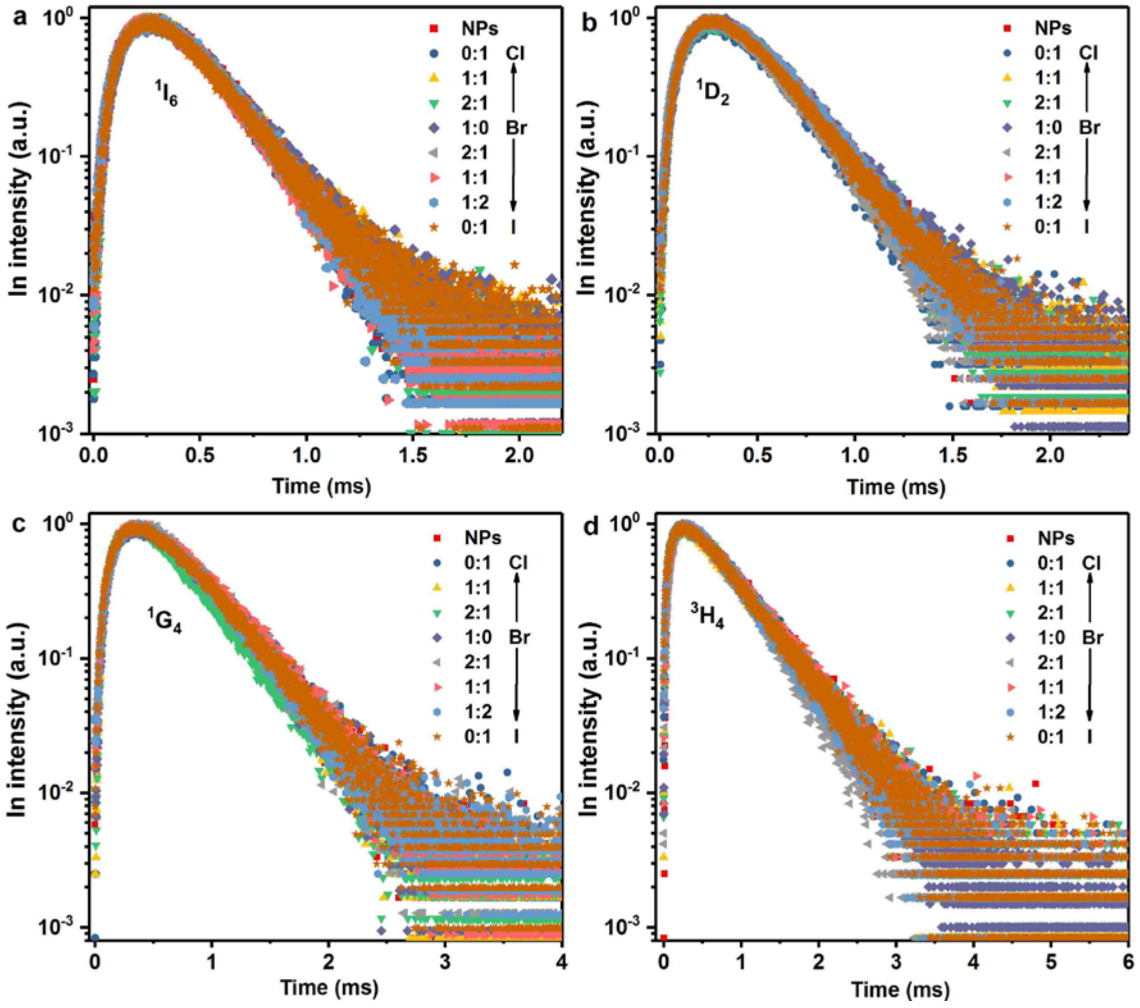

Supplementary Figure 11. UCL decays from **a** $^1I_6$, **b** $^1D_2$, **c** $^1G_4$, and **d** $^3H_4$ of Tm^{3+} by monitoring the Tm^{3+} emissions at 347, 362, 483, and 792 nm, respectively, in pure $LiYbF_4$:0.5%Tm^{3+}@$LiYF_4$ core/shell NPs and NPs-sensitized $CsPbX_3$ PeQDs with varying halide compositions under 980 nm excitation. The concentrations of the NPs and PeQDs are 1 and 0.2 mg mL^{-1}, respectively. All UCL decays are nearly identical for each level of Tm^{3+} and are independent of the composition and absorption of PeQDs, thus providing a solid evidence for the radiative energy transfer from the NPs to PeQDs.

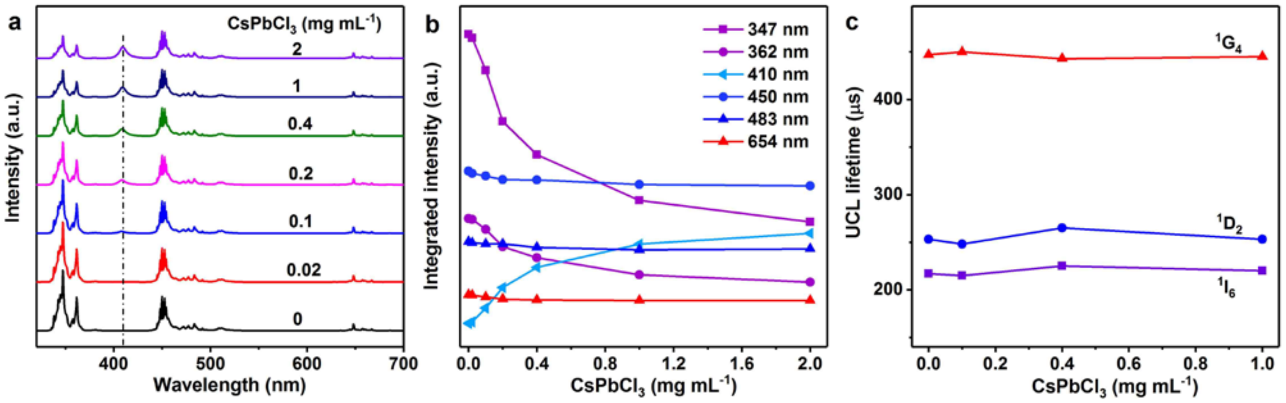

Supplementary Figure 12. a $CsPbCl_3$ concentration-dependent UCL spectra for NPs-sensitized $CsPbCl_3$ PeQDs with NPs concentration of 1 mg mL^{-1} under 980 nm CW diode laser excitation at a power density of 50 W cm^{-2}, showing a gradual increase in $CsPbCl_3$ emission at the expense of the UV emissions of Tm^{3+}. **b** Integrated intensities of the Tm^{3+} emissions at 347 nm ($^1I_6 \rightarrow {}^3F_4$), 362 nm ($^1D_2 \rightarrow {}^3H_6$), 450 nm ($^1D_2 \rightarrow {}^3F_4$), 483 nm ($^1G_4 \rightarrow {}^3H_6$) and 648 nm ($^1G_4 \rightarrow {}^3F_4$), and the $CsPbCl_3$ emission at 410 nm versus the $CsPbCl_3$ concentration, as obtained from (**a**). **c** UCL lifetimes of $^1I_6$, $^1D_2$, and $^1G_4$ of Tm^{3+} in NPs-sensitized $CsPbCl_3$ PeQDs versus the $CsPbCl_3$ concentration. The distinct UCL evolutions between the UV (362 nm) and blue (450 nm) emissions from $^1D_2$ and the nearly unchanged UCL lifetimes of Tm^{3+} with increasing the $CsPbCl_3$ concentration, demonstrate that the energy transfer from the NPs to $CsPbCl_3$ PeQDs is dictated by a radiative reabsorption process instead of a non-radiative FRET process.

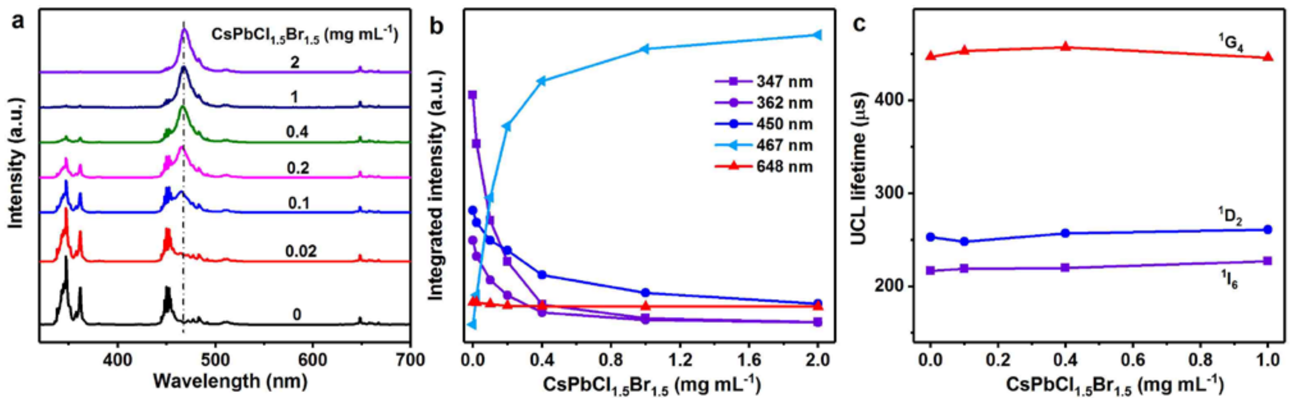

Supplementary Figure 13. a $CsPbCl_{1.5}Br_{1.5}$ concentration-dependent UCL spectra for NPs-sensitized $CsPbCl_{1.5}Br_{1.5}$ PeQDs with NPs concentration of 1 mg mL^{-1} under 980 nm CW diode laser excitation at a power density of 50 W cm^{-2}, showing a gradual increased $CsPbCl_{1.5}Br_{1.5}$ emission at the expense of the UV and blue emissions of Tm^{3+}. **b** Integrated intensities of the Tm^{3+} emissions at 347 nm ($^1I_6 \rightarrow {}^3F_4$), 362 nm ($^1D_2 \rightarrow {}^3H_6$), 450 nm ($^1D_2 \rightarrow {}^3F_4$) and 648 nm ($^1G_4 \rightarrow {}^3F_4$), and the $CsPbCl_{1.5}Br_{1.5}$ emission at 467 nm versus the $CsPbCl_{1.5}Br_{1.5}$ concentration, as obtained from (**a**). **c** UCL lifetimes of $^1I_6$, $^1D_2$, and $^1G_4$ of Tm^{3+} in NPs-sensitized $CsPbCl_{1.5}Br_{1.5}$ PeQDs versus the $CsPbCl_{1.5}Br_{1.5}$ concentration. The distinct UCL evolutions between the UV (362 nm) and blue (450 nm) emissions from $^1D_2$ and the nearly unchanged UCL lifetimes of Tm^{3+} with increasing the $CsPbCl_{1.5}Br_{1.5}$ concentration, demonstrate that the energy transfer from the NPs to $CsPbCl_{1.5}Br_{1.5}$ PeQDs is a radiative energy transfer process instead of a non-radiative FRET process.

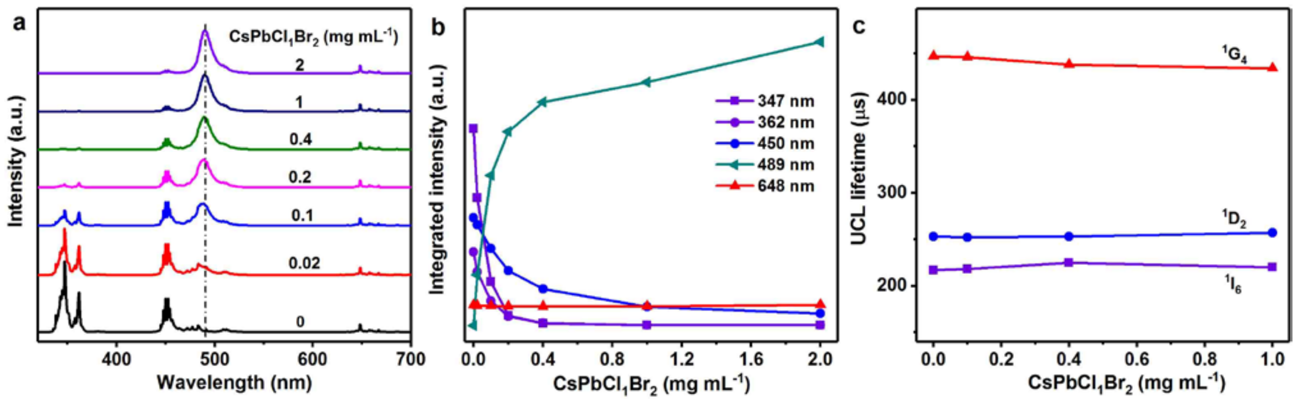

Supplementary Figure 14. a $CsPbCl_1Br_2$ concentration-dependent UCL spectra for NPs-sensitized $CsPbCl_1Br_2$ PeQDs with NPs concentration of 1 mg mL^{-1} under 980 nm CW diode laser excitation at a power density of 50 W cm^{-2}, showing a gradual increase in $CsPbCl_1Br_2$ emission at the expense of the UV and blue emissions of Tm^{3+}. **b** Integrated intensities of the Tm^{3+} emissions at 347 nm ($^1I_6 \rightarrow {}^3F_4$), 362 nm ($^1D_2 \rightarrow {}^3H_6$), 450 nm ($^1D_2 \rightarrow {}^3F_4$) and 648 nm ($^1G_4 \rightarrow {}^3F_4$), and the $CsPbCl_1Br_2$ emission at 489 nm versus the $CsPbCl_1Br_2$ concentration, as obtained from (**a**). **c** UCL lifetimes of $^1I_6$, $^1D_2$, and $^1G_4$ of Tm^{3+} in NPs-sensitized $CsPbCl_1Br_2$ PeQDs versus the $CsPbCl_1Br_2$ concentration. The distinct UCL evolutions between the UV (362 nm) and blue (450 nm) emissions from $^1D_2$ and the nearly unchanged UCL lifetimes of Tm^{3+} with increasing the $CsPbCl_1Br_2$ concentration, reveal that the energy transfer from the NPs to $CsPbCl_1Br_2$ PeQDs is mediated by a radiative reabsorption process rather than a non-radiative FRET process.

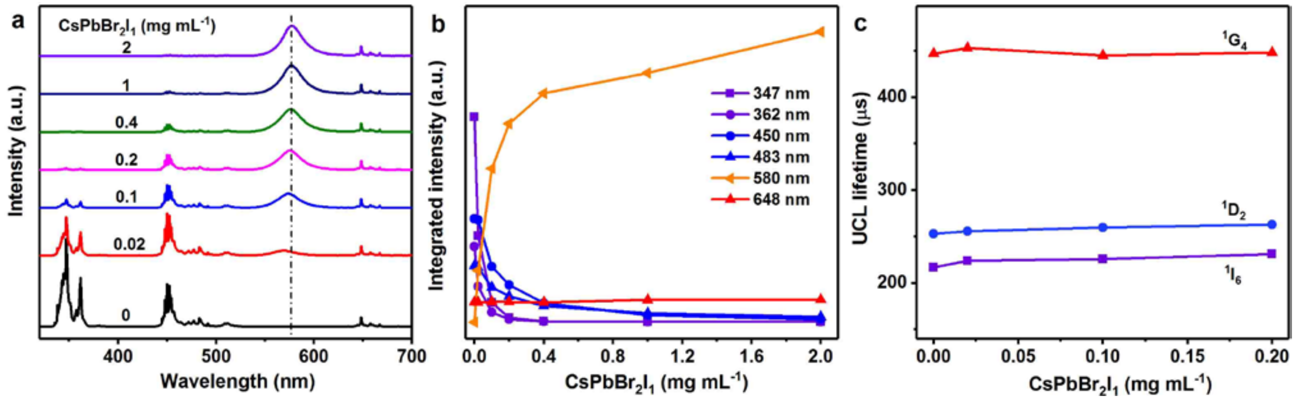

Supplementary Figure 15. **a** $CsPbBr_2I_1$ concentration-dependent UCL spectra for NPs-sensitized $CsPbBr_2I_1$ PeQDs with NPs concentration of 1 mg mL^{-1} under 980 nm CW diode laser excitation at a power density of 50 W cm^{-2}, showing a stepwise increased $CsPbBr_2I_1$ emission at the expense of the UV and blue emissions of Tm^{3+}. **b** Integrated intensities of the Tm^{3+} emissions at 347 nm ($^1I_6\rightarrow{}^3F_4$), 362 nm ($^1D_2\rightarrow{}^3H_6$), 450 nm ($^1D_2\rightarrow{}^3F_4$), 483 nm ($^1G_4\rightarrow{}^3H_6$) and 648 nm ($^1G_4\rightarrow{}^3F_4$), and the $CsPbBr_2I_1$ emission at 580 nm versus the $CsPbBr_2I_1$ concentration, as obtained from (**a**). **c** UCL lifetimes of $^1I_6$, $^1D_2$, and $^1G_4$ of Tm^{3+} in NPs-sensitized $CsPbBr_2I_1$ PeQDs versus the $CsPbBr_2I_1$ concentration. The distinct UCL evolutions between the UV (362 nm) and blue (450 nm) emissions from $^1D_2$ and between the blue (483 nm) and red (648 nm) emissions from $^1G_4$, and the nearly unchanged UCL lifetimes of Tm^{3+} with increasing the $CsPbBr_2I_1$ concentration, demonstrate that the energy transfer from the NPs to $CsPbBr_2I_1$ PeQDs is a radiative energy transfer process instead of a non-radiative FRET process.

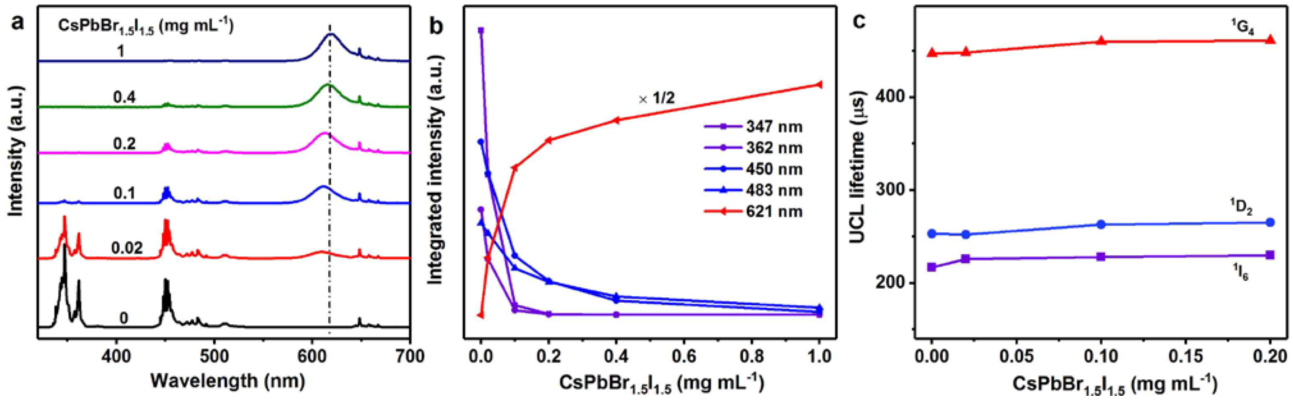

Supplementary Figure 16. a $CsPbBr_{1.5}I_{1.5}$ concentration-dependent UCL spectra for NPs-sensitized $CsPbBr_{1.5}I_{1.5}$ PeQDs with NPs concentration of 1 mg mL^{-1} under 980 nm CW diode laser excitation at a power density of 50 W cm^{-2}, showing a gradual increase in $CsPbBr_{1.5}I_{1.5}$ emission at the expense of the UV and blue emissions of Tm^{3+}. **b** Integrated intensities of the Tm^{3+} emissions at 347 nm ($^1I_6 \rightarrow ^3F_4$), 362 nm ($^1D_2 \rightarrow ^3H_6$), 450 nm ($^1D_2 \rightarrow ^3F_4$) and 483 nm ($^1G_4 \rightarrow ^3H_6$), and the $CsPbBr_{1.5}I_{1.5}$ emission at 621 nm versus the $CsPbBr_{1.5}I_{1.5}$ concentration, as obtained from (**a**). **c** UCL lifetimes of $^1I_6$, $^1D_2$, and $^1G_4$ of Tm^{3+} in NPs-sensitized $CsPbBr_{1.5}I_{1.5}$ PeQDs versus the $CsPbBr_{1.5}I_{1.5}$ concentration. The distinct UCL evolutions between the UV (362 nm) and blue (450 nm) emissions from $^1D_2$ and between the blue (483 nm) and red (648 nm) emissions from $^1G_4$, and the nearly unchanged UCL lifetimes of Tm^{3+} with increasing the $CsPbBr_{1.5}I_{1.5}$ concentration, demonstrate that the energy transfer from the NPs to $CsPbBr_{1.5}I_{1.5}$ PeQDs is governed by a radiative reabsorption process instead of a non-radiative FRET process.

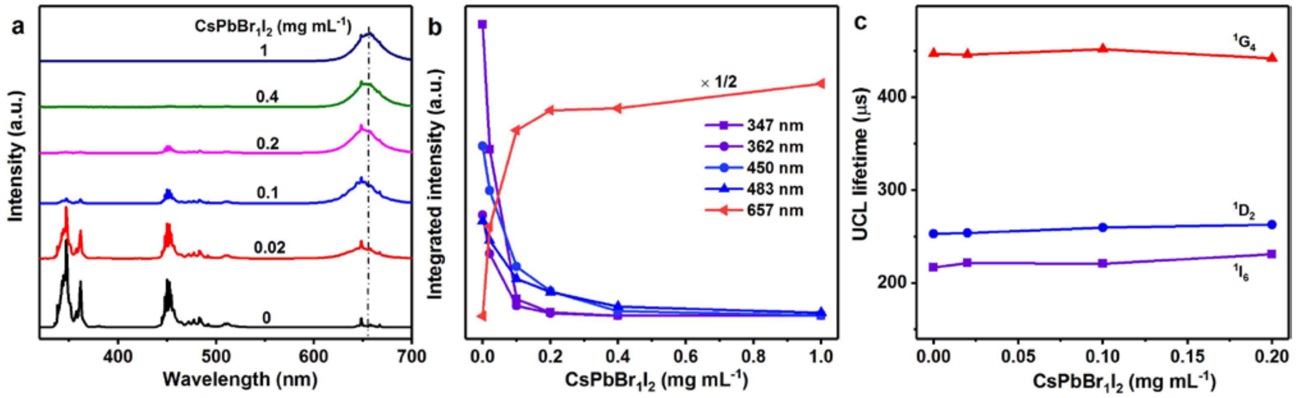

Supplementary Figure 17. a $CsPbBr_1I_2$ concentration-dependent UCL spectra for NPs-sensitized $CsPbBr_1I_2$ PeQDs with NPs concentration of 1 mg mL^{-1} under 980 nm CW diode laser excitation at a power density of 50 W cm^{-2}, showing a stepwise increased $CsPbBr_1I_2$ emission at the expense of the UV, blue, and red emissions of Tm^{3+}. **b** Integrated intensities of the Tm^{3+} emissions at 347 nm ($^1I_6 \rightarrow {}^3F_4$), 362 nm ($^1D_2 \rightarrow {}^3H_6$), 450 nm ($^1D_2 \rightarrow {}^3F_4$) and 483 nm ($^1G_4 \rightarrow {}^3H_6$), and the $CsPbBr_1I_2$ emission at 657 nm versus the $CsPbBr_1I_2$ concentration, as obtained from (**a**). **c** UCL lifetimes of $^1I_6$, $^1D_2$, and $^1G_4$ of Tm^{3+} in NPs-sensitized $CsPbBr_1I_2$ PeQDs versus the $CsPbBr_1I_2$ concentration. The distinct UCL evolutions between the UV (362 nm) and blue (450 nm) emissions from $^1D_2$ and between the blue (483 nm) and red (648 nm) emissions from $^1G_4$, and the nearly unchanged UCL lifetimes of Tm^{3+} with increasing the $CsPbBr_1I_2$ concentration, demonstrate that the energy transfer from the NPs to $CsPbBr_1I_2$ PeQDs is a radiative energy transfer process instead of a non-radiative FRET process.

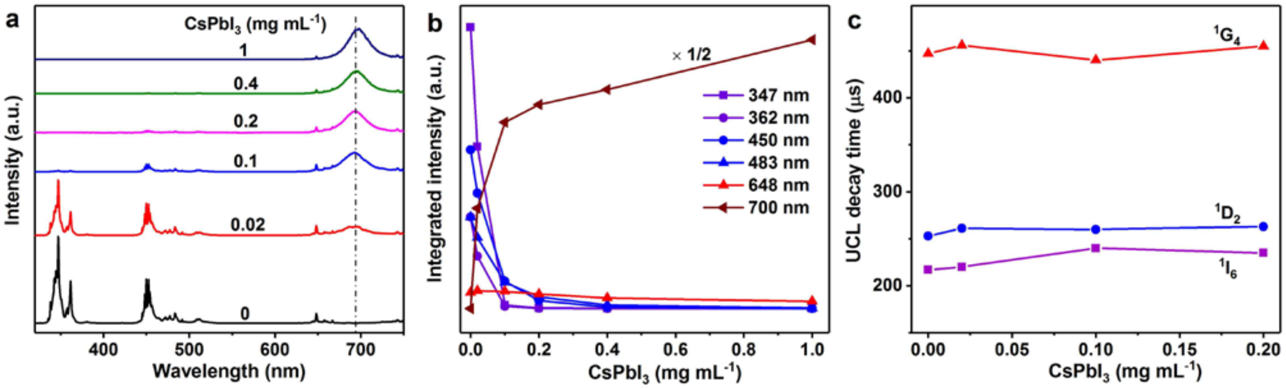

Supplementary Figure 18. a $CsPbI_3$ concentration-dependent UCL spectra for NPs-sensitized $CsPbI_3$ PeQDs with NPs concentration of 1 mg mL^{-1} under 980 nm CW diode laser excitation at a power density of 50 W cm^{-2}, showing a gradual increase in $CsPbI_3$ emission at the expense of the UV, blue, and red emissions of Tm^{3+}. **b** Integrated intensities of the Tm^{3+} emissions at 347 nm ($^1I_6 \rightarrow {}^3F_4$), 362 nm ($^1D_2 \rightarrow {}^3H_6$), 450 nm ($^1D_2 \rightarrow {}^3F_4$), 483 nm ($^1G_4 \rightarrow {}^3H_6$) and 648 nm ($^1G_4 \rightarrow {}^3F_4$), and the $CsPbI_3$ emission at 700 nm versus the $CsPbI_3$ concentration, as obtained from (**a**). **c** UCL lifetimes of $^1I_6$, $^1D_2$, and $^1G_4$ of Tm^{3+} in NPs-sensitized $CsPbI_3$ PeQDs versus the $CsPbI_3$ concentration. The distinct UCL evolutions between the UV (362 nm) and blue (450 nm) emissions from $^1D_2$ and between the blue (483 nm) and red (648 nm) emissions from $^1G_4$, and the nearly unchanged UCL lifetimes of Tm^{3+} with increasing $CsPbI_3$ concentration, demonstrate that the energy transfer from the NPs to $CsPbI_3$ PeQDs is radiative energy transfer rather than non-radiative FRET.

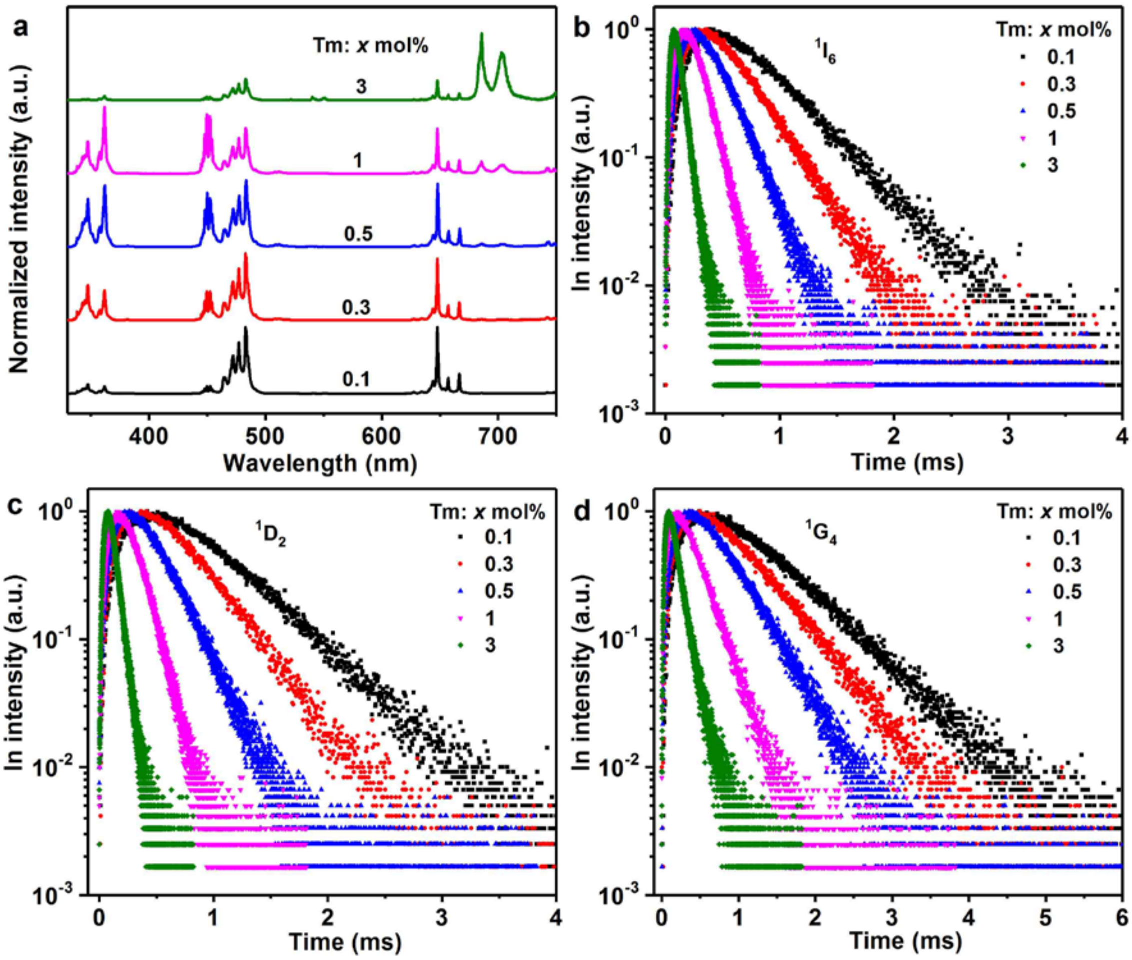

Supplementary Figure 19. a UCL spectra for $LiYbF_4$:x%Tm^{3+}@$LiYF_4$ core/shell NPs with varying Tm^{3+} concentration under 980 nm CW diode laser excitation at a power density of 50 W cm^{-2}. The corresponding UCL decays from **b** $^1I_6$, **c** $^1D_2$, and **d** $^1G_4$ of Tm^{3+} by monitoring the Tm^{3+} emissions at 347, 362, and 483 nm, respectively. It was observed that the UV and blue emissions of Tm^{3+} decreased drastically when the Tm^{3+} concentration increased to 3 mol%, while the emission peaks at 685 and 703 nm which can be assigned to $^3F_{2,3}\rightarrow{}^3H_6$ transitions of Tm^{3+} intensified gradually with increasing the Tm^{3+} concentration. This can be ascribed to a synergic effect of the aggravated cross relaxation among Tm^{3+} ions and the increased energy back transfer from Yb^{3+} to Tm^{3+} arising from the decreased distance between Tm^{3+} and Yb^{3+} ions at higher Tm^{3+} concentrations, as also evidenced by the decreased UCL lifetimes of Tm^{3+} with increasing the Tm^{3+} concentration. By single-exponential fitting to the decay curves, the UCL lifetimes of $^1I_6$, $^1D_2$, and $^1G_4$ of Tm^{3+} were determined to decrease from 473, 553, and 803 μs (0.1 mol%) to 60, 61, and 119 μs (3 mol%), respectively.

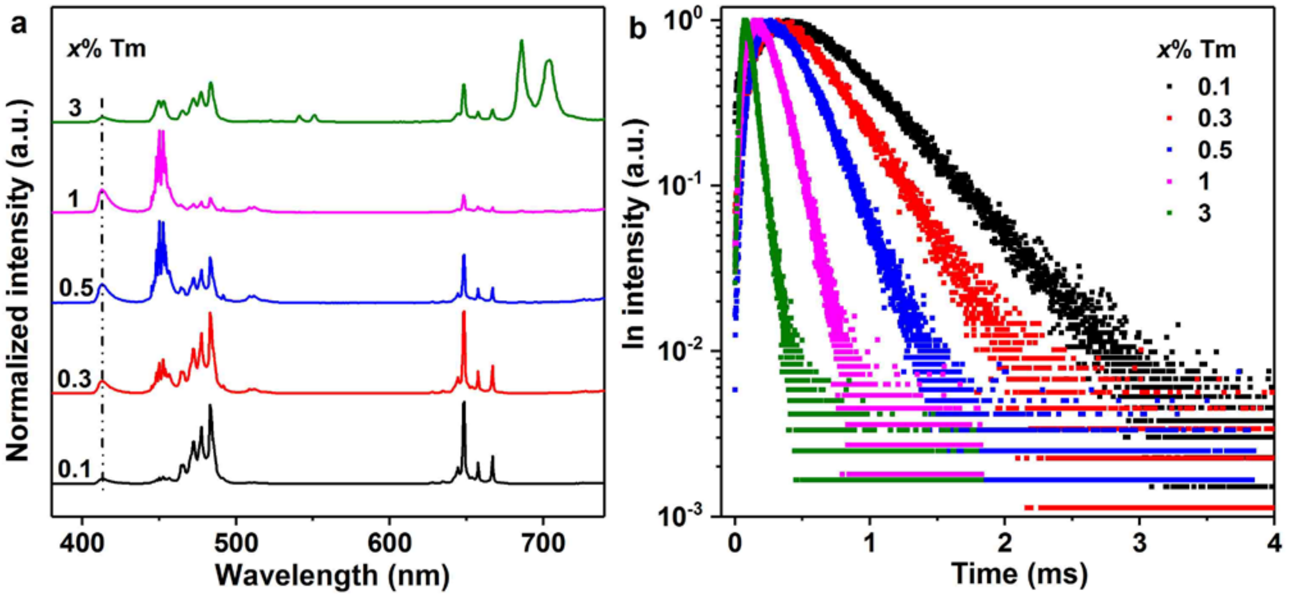

Supplementary Figure 20. a UCL spectra for NPs-sensitized $CsPbCl_3$ PeQDs with varying Tm^{3+} concentration in the NPs under 980 nm CW diode laser excitation at a power density of 50 W cm^{-2}. **b** The corresponding UCL decays from band-edge excitons of $CsPbCl_3$ by monitoring their emission at 410 nm under 980 nm excitation, showing tunable lifetimes from 494 μs to 61 μs as the Tm^{3+} concentration increased from 0.1 mol% to 3 mol%. The rising edge in the initial stage of the decay curves reflects the slow population of the PeQDs excited state from the long-lived Tm^{3+} excited state, as a result of radiative energy transfer from the NPs to $CsPbCl_3$ PeQDs.

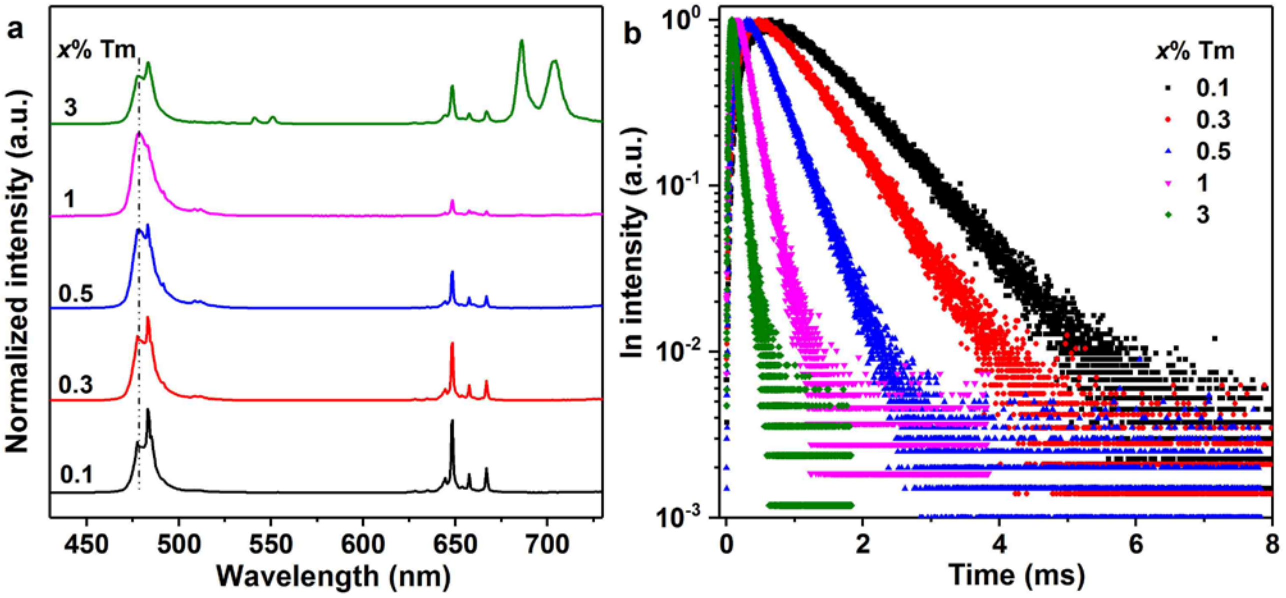

Supplementary Figure 21. a UCL spectra for NPs-sensitized $CsPbCl_{1.5}Br_{1.5}$ PeQDs with varying Tm^{3+} concentration in the NPs under 980 nm CW diode laser excitation at a power density of 50 W cm^{-2}. **b** The corresponding UCL decays from band-edge excitons of $CsPbCl_{1.5}Br_{1.5}$ by monitoring their emission at 467 nm under 980 nm excitation, showing tunable lifetimes from 978 μs to 79 μs as the Tm^{3+} concentration increased from 0.1 mol% to 3 mol%. The rising edge in the initial stage of the decay curves reflects the slow population of the PeQDs excited state from the long-lived Tm^{3+} excited state, as a result of radiative energy transfer from the NPs to $CsPbCl_{1.5}Br_{1.5}$ PeQDs.

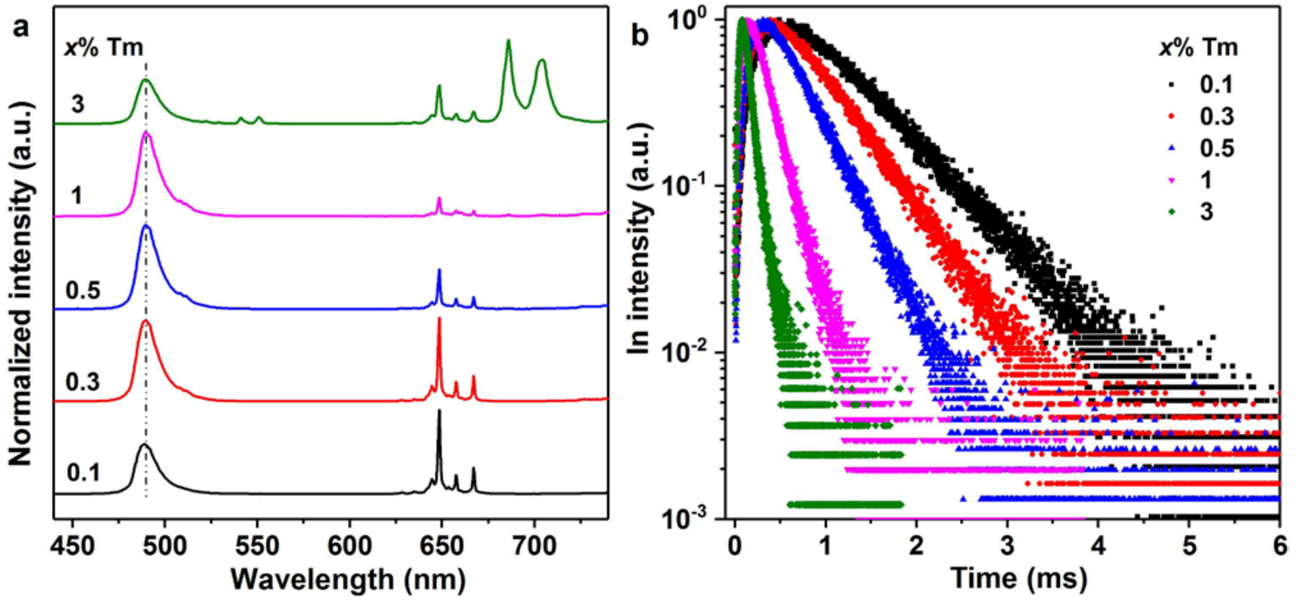

Supplementary Figure 22. a UCL spectra for NPs-sensitized $CsPbCl_1Br_2$ PeQDs with varying Tm^{3+} concentration in the NPs under 980 nm CW diode laser excitation at a power density of 50 W cm^{-2}. **b** The corresponding UCL decays from band-edge excitons of $CsPbCl_1Br_2$ by monitoring their emission at 489 nm under 980 nm excitation, showing tunable lifetimes from 775 μs to 81 μs as the Tm^{3+} concentration increased from 0.1 mol% to 3 mol%. The rising edge in the initial stage of the decay curves reflects the slow population of the PeQDs excited state from the long-lived Tm^{3+} excited state, as a result of radiative energy transfer from the NPs to $CsPbCl_1Br_2$ PeQDs.

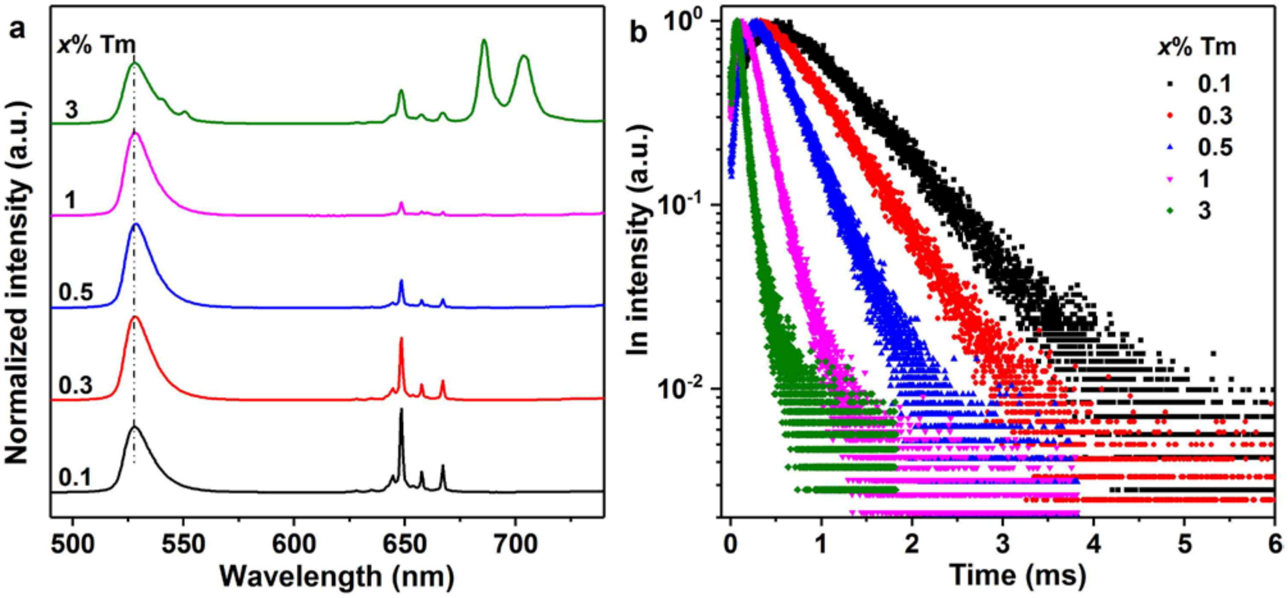

Supplementary Figure 23. a UCL spectra for NPs-sensitized $CsPbBr_3$ PeQDs with varying Tm^{3+} concentration in the NPs under 980 nm CW diode laser excitation at a power density of 50 W cm^{-2}. **b** The corresponding UCL decays from band-edge excitons of $CsPbBr_3$ by monitoring their emission at 520 nm under 980 nm excitation, showing tunable lifetimes from 794 μs to 81 μs as the Tm^{3+} concentration increased from 0.1 mol% to 3 mol%. The rising edge in the initial stage of the decay curves reflects the slow population of the PeQDs excited state from the long-lived Tm^{3+} excited state, as a result of radiative energy transfer from the NPs to $CsPbBr_3$ PeQDs.

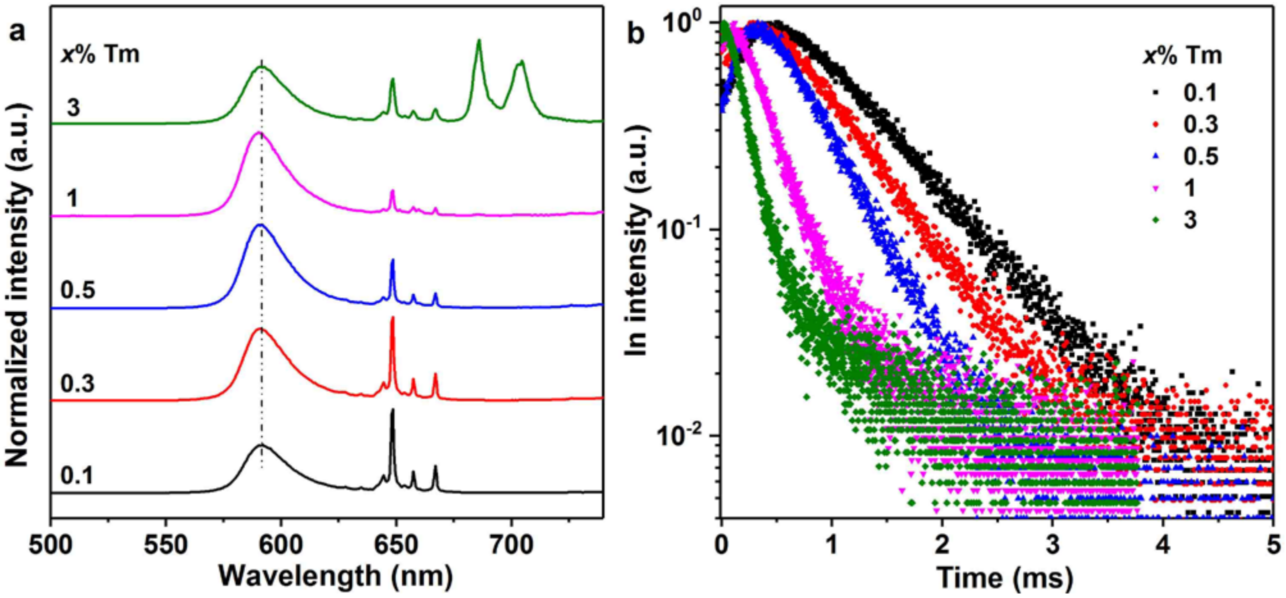

Supplementary Figure 24. a UCL spectra for NPs-sensitized $CsPbBr_2I_1$ PeQDs with varying Tm^{3+} concentration in the NPs under 980 nm CW diode laser excitation at a power density of 50 W cm^{-2}. **b** The corresponding UCL decays from band-edge excitons of $CsPbBr_2I_1$ by monitoring their emission at 580 nm under 980 nm excitation, showing tunable lifetimes from 742 μs to 198 μs as the Tm^{3+} concentration increased from 0.1 mol% to 3 mol%. The rising edge in the initial stage of the decay curves reflects the slow population of the PeQDs excited state from the long-lived Tm^{3+} excited state, as a result of radiative energy transfer from the NPs to $CsPbBr_2I_1$ PeQDs. Note that the UCL signal in the beginning of the decay curves arising from two-photon absorption of $CsPbBr_2I_1$ under 980 nm nanosecond-pulsed laser excitation had been deleted to guarantee the purity of the UCL signal from energy transfer.

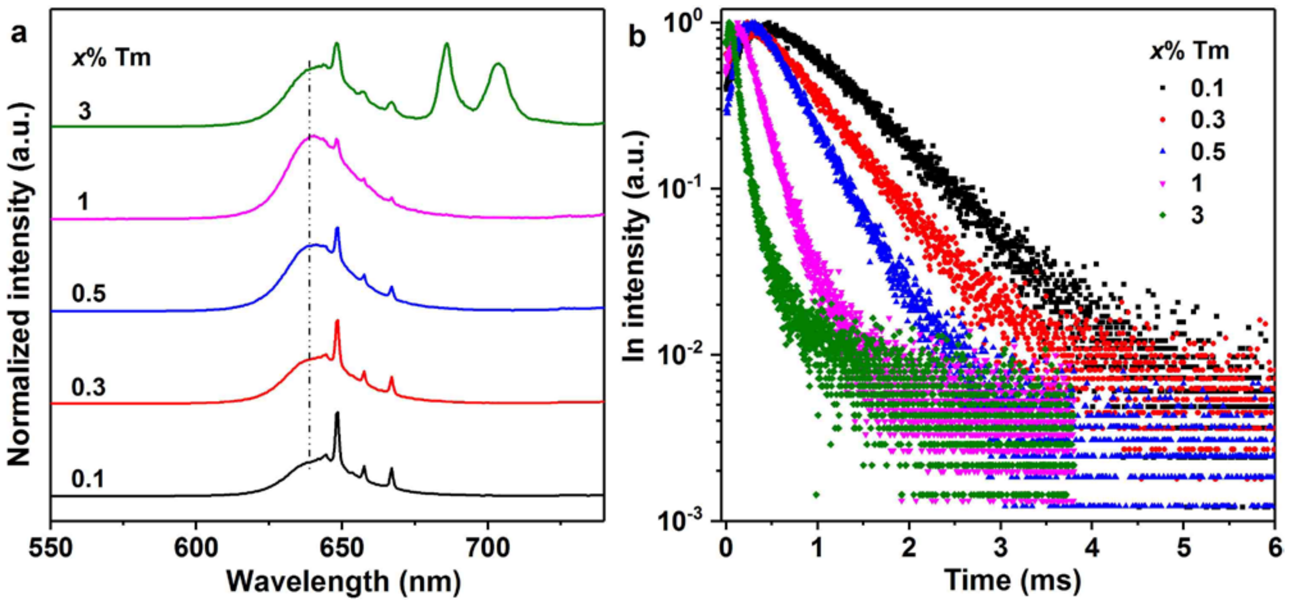

Supplementary Figure 25. a UCL spectra for NPs-sensitized $CsPbBr_{1.5}I_{1.5}$ PeQDs with varying Tm^{3+} concentration in the NPs under 980 nm CW diode laser excitation at a power density of 50 W cm^{-2}. **b** The corresponding UCL decays from band-edge excitons of $CsPbBr_{1.5}I_{1.5}$ by monitoring their emission at 630 nm under 980 nm excitation, showing tunable lifetimes from 840 μs to 100 μs as the Tm^{3+} concentration increased from 0.1 mol% to 3 mol%. The red shift in the emission band of $CsPbBr_{1.5}I_{1.5}$ QDs relative to that in Supplementary Fig. 15 is attributed to their increased particle size during their storage for one month. The rising edge in the initial stage of the decay curves reflects the slow population of the PeQDs excited state from the long-lived Tm^{3+} excited state, as a result of radiative energy transfer from the NPs to $CsPbBr_{1.5}I_{1.5}$ PeQDs. Note that the UCL signal in the beginning of the decay curves arising from two-photon absorption of $CsPbBr_{1.5}I_{1.5}$ under 980 nm nanosecond-pulsed laser excitation had been deleted to guarantee the purity of the UCL signal from energy transfer.

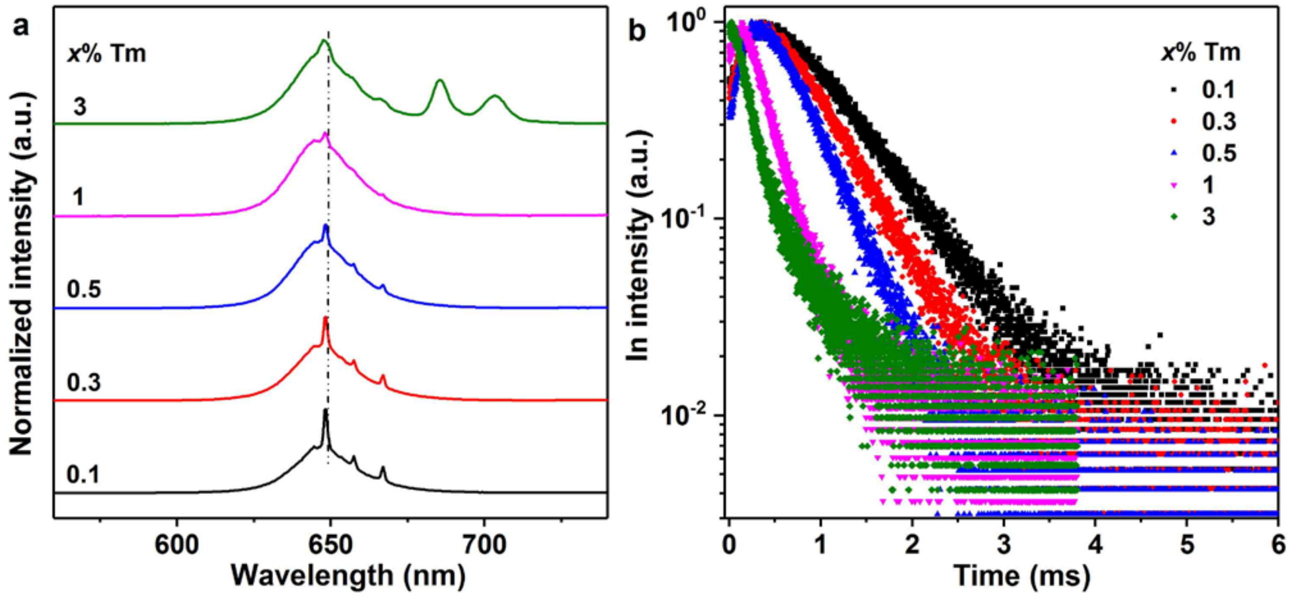

Supplementary Figure 26. a UCL spectra for NPs-sensitized $CsPbBr_1I_2$ PeQDs with varying Tm^{3+} concentration in the NPs under 980 nm CW diode laser excitation at a power density of 50 W cm^{-2}. **b** The corresponding UCL decays from band-edge excitons of $CsPbBr_1I_2$ by monitoring their emission at 657 nm under 980 nm excitation, showing tunable lifetimes from 727 μs to 243 μs as the Tm^{3+} concentration increased from 0.1 mol% to 3 mol%. The rising edge in the initial stage of the decay curves reflects the slow population of the PeQDs excited state from the long-lived Tm^{3+} excited state, as a result of radiative energy transfer from the NPs to $CsPbBr_1I_2$ PeQDs. Note that the UCL signal in the beginning of the decay curves arising from two-photon absorption of $CsPbBr_1I_2$ under 980 nm nanosecond-pulsed laser excitation had been deleted to guarantee the purity of the UCL signal from energy transfer.

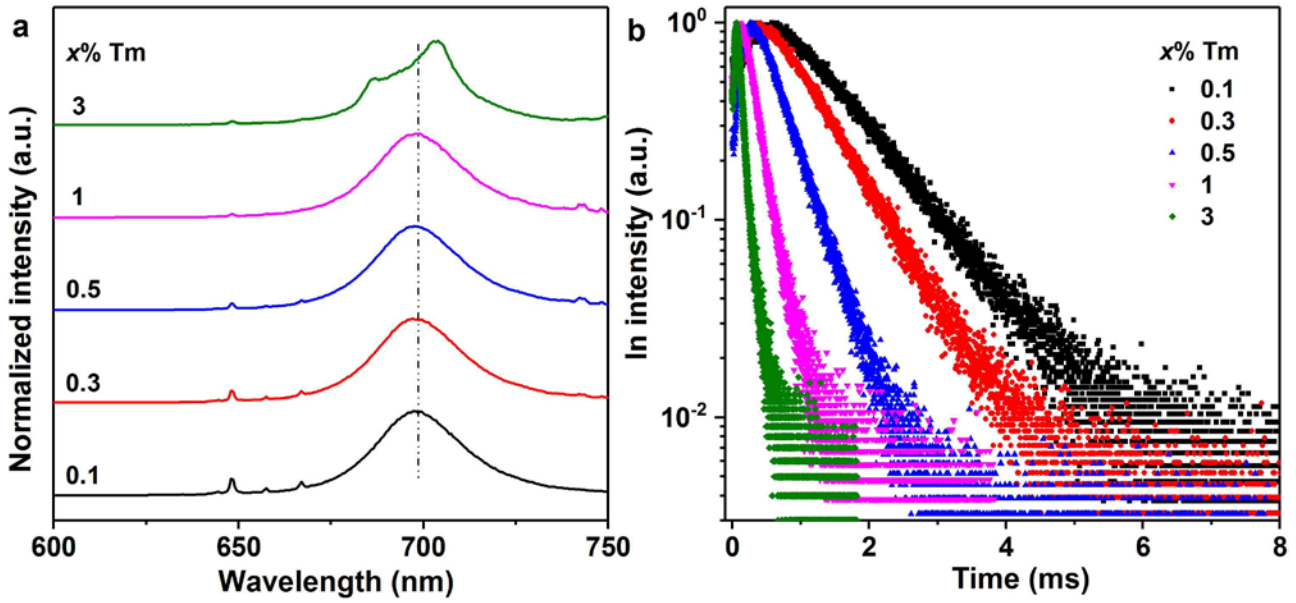

Supplementary Figure 27. a UCL spectra for NPs-sensitized $CsPbI_3$ PeQDs with varying Tm^{3+} concentration in the NPs under 980 nm CW diode laser excitation at a power density of 50 W cm^{-2}. **b** The corresponding UCL decays from band-edge excitons of $CsPbI_3$ by monitoring their emission at 700 nm under 980 nm excitation, showing tunable lifetimes from 1.053 ms to 80 μs as the Tm^{3+} concentration increased from 0.1 mol% to 3 mol%. The rising edge in the initial stage of the decay curves reflects the slow population of the PeQDs excited state from the long-lived Tm^{3+} excited state, as a result of radiative energy transfer from the NPs to $CsPbI_3$ PeQDs. Note that the UCL signal in the beginning of the decay curves arising from two-photon absorption of $CsPbI_3$ under 980 nm nanosecond-pulsed laser excitation had been deleted to guarantee the purity of the UCL signal from energy transfer.

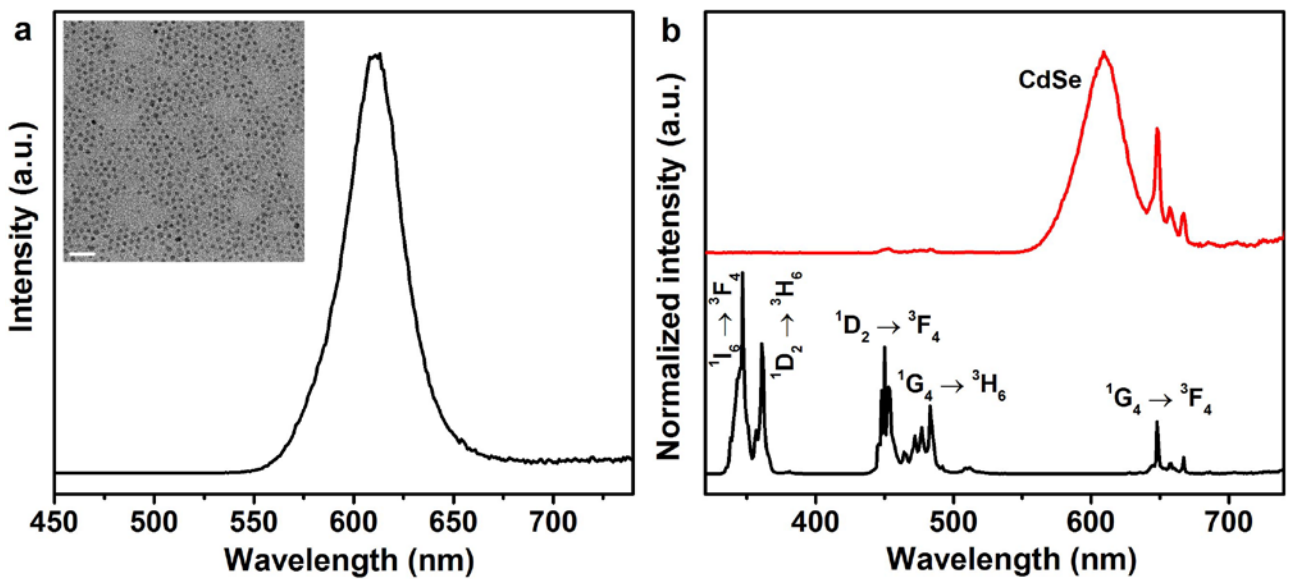

Supplementary Figure 28. a PL spectrum of CdSe quantum dots (QDs) under UV excitation at 365 nm. The inset shows the TEM image of CdSe QDs, indicating a mean size of ~4 nm. The scale bar represents 20 nm. **b** UCL spectra of $LiYbF_4$:0.5%Tm^{3+}@$LiYF_4$ core/shell NPs and the NPs-sensitized CdSe QDs under 980 nm CW diode laser excitation at a power density of 20 W cm^{-2}. CdSe QDs were synthesized via a hot-injection method as previously reported by C. L. Yan *et al*. (Supplementary Ref. 4). The measurement sample was prepared by dispersing the NPs and the QDs in cyclohexane with concentrations of 1 and 2 mg mL^{-1}, respectively. Under 980 nm excitation, the NPs-sensitized QDs displayed characteristic QDs exciton emission at 610 nm, while the UV and blue emissions of Tm^{3+} from the NPs were completely quenched, as a result of radiative energy transfer from the NPs to the QDs.

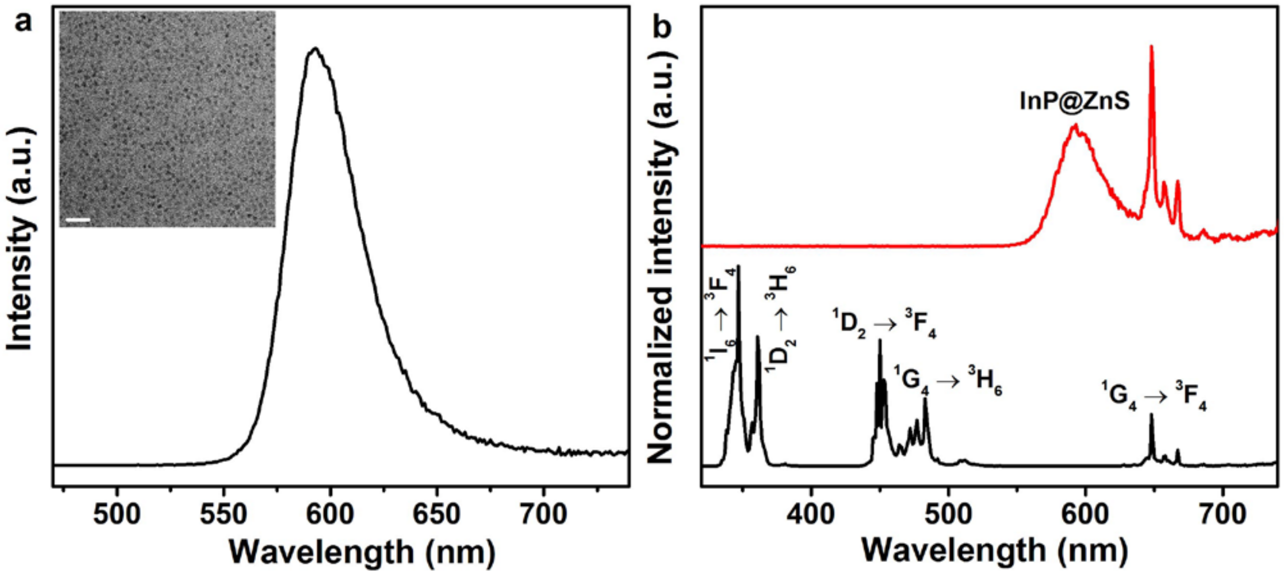

Supplementary Figure 29. a PL spectrum of InP@ZnS core/shell QDs under UV excitation at 365 nm. The inset shows the TEM image of InP@ZnS QDs, indicating a mean size of ~3 nm. The scale bar represents 20 nm. **b** UCL spectra of $LiYbF_4$:0.5%Tm^{3+}@$LiYF_4$ core/shell NPs and the NPs-sensitized InP@ZnS QDs under 980 nm CW diode laser excitation at a power density of 20 W cm^{-2}. InP@ZnS core/shell QDs were synthesized via a hot-injection method as previously reported by M. D. Tessier *et al*. (Supplementary Ref. 5). The measurement sample was prepared by dispersing the NPs and the QDs in cyclohexane with concentrations of 1 and 2 mg mL^{-1}, respectively. Under 980 nm excitation, the NPs-sensitized QDs displayed characteristic QDs exciton emission at 590 nm, while the UV and blue emissions of Tm^{3+} from the NPs were completely quenched, as a result of radiative energy transfer from the NPs to the QDs.

Supplementary References

1. Zou, Q., *et al*. Cooperative and non-cooperative sensitization upconversion in lanthanide-doped $LiYbF_4$ nanoparticles. *Nanoscale* **9**, 6521-6528 (2017).
2. Boyer, J. & van Veggel, F. Absolute quantum yield measurements of colloidal $NaYF_4$:Er^{3+},Yb^{3+} upconverting nanoparticles. *Nanoscale* **2**, 1417-1419 (2010).
3. Würth, C., Grabolle, M., Pauli, J., Spieles, M. & Resch-Genger, U. Relative and absolute determination of fluorescence quantum yields of transparent samples. *Nat. Protoc.* **8**, 1535-1550 (2013).
4. Yan, C., Dadvand, A., Rosei, F. & Perepichka, D. Near-IR photoresponse in new up-converting CdSe/$NaYF_4$:Yb,Er nanoheterostructures. *J. Am. Chem. Soc.* **132**, 8868-8869 (2010).
5. Tessier, M., Dupont, D., Nolf, K., Roo, J. & Hens, Z. Economic and size-tunable synthesis of InP/ZnE (E = S, Se) colloidal quantum dots. *Chem. Mater.* **27**, 4893-4898 (2015).
